# Improved genetic resolution for linkage mapping of resistance to potato wart in monoparental dihaploids with potential diagnostic value in tetraploid potato varieties

**DOI:** 10.1007/s00122-018-3172-9

**Published:** 2018-08-29

**Authors:** Annette Bartkiewicz, Friederike Chilla, Diro Terefe-Ayana, Jens Lübeck, Josef Strahwald, Eckhard Tacke, Hans-Reinhard Hofferbert, Kerstin Flath, Marcus Linde, Thomas Debener

**Affiliations:** 1 Institute of Plant Genetics, Department of Molecular Plant Breeding, Leibniz University Hannover, Hannover, Germany; 2 Westhoff, Südlohn-Oeding, Germany; 3 SaKa Pflanzenzucht GmbH & Co. KG, Windeby, Germany; 4 Böhm-Nordkartoffel Agrarproduktion GmbH & Co. OHG, Ebstorf, Germany; 5 Julius Kühn-Institut, Kleinmachnow, Germany; 6 DHD-Consulting GmbH, Hildesheim, Germany

## Abstract

**Key message:**

We achieved improved mapping resolution of the major wart resistance locus Xla-TNL *containing also Sen1* in a dihaploid population using SNP data and developed additional markers with diagnostic value in tetraploid varieties.

**Abstract:**

We analyzed a segregating monoparental dihaploid potato population comprising 215 genotypes derived from a tetraploid variety that is highly resistant to *Synchytrium endobioticum* pathotypes 18 and 6. The clear bimodal segregation for both pathotypes indicated that a major dominant resistance factor in a simplex allele configuration was present in the tetraploid donor genotype. Compared to that in previous analyses of the same tetraploid donor in conventional crosses with susceptible tetraploid genotypes, a segregation pattern with a reduced genetic complexity of resistance in dihaploids was observed here. Using the 12.8 k SolCAP SNP array, we mapped a resistance locus to the Xla-TNL region *containing also Sen1* on potato chromosome 11. The improved mapping resolution provided by the monoparental dihaploids allowed for the localization of the genes responsible for the resistance to both pathotypes in an interval spanning less than 800 kbp on the reference genome. Furthermore, we identified eight molecular markers segregating without recombination to pathotype 18 and pathotype 6 resistance. Also, two developed markers display improved diagnostic properties in an independent panel of tetraploid varieties. Overall, our data provide the highest resolution mapping of wart resistance genes at the Xla-TNL locus thus far.

**Electronic supplementary material:**

The online version of this article (10.1007/s00122-018-3172-9) contains supplementary material, which is available to authorized users.

## Introduction

The obligate biotrophic soil-borne fungus *Synchytrium endobioticum* (Schilb.) Perc. belongs to the Chytridiomycetes class and is the causal agent of potato wart disease. This pathogen is classified as an A2 quarantine pest by the European and Mediterranean Plant Protection Organization (EPPO 2004) and is globally distributed (Obidiegwu et al. 2014). This pathogen can infect potato tubers, stolons and stems and causes yield losses of up to 50–100% (Hampson 1993; Melnik 1998). The typical symptoms of potato wart include the formation of cauliflower-like irregular galls that vary in size and shape (Franc 2007). The wart tissue consists of hypertrophic, tumor-like dividing cells with thin-walled summer sori and thick-walled winter sori that can survive in soil for up to 30–40 years (Laidlaw 1985). Controlling this disease is very difficult due to the limited ability of fungicides to access the winter sporangia. Furthermore, chemicals that are effective against potato wart disease are also phytotoxic (Hampson 1977; Gunacti and Erkiliç 2013). Strict phytosanitary measures and the breeding and cultivation of resistant cultivars are the only feasible strategies for controlling potato wart disease. More than 40 different pathotypes of this pathogen have been reported (Baayen et al. 2006; Çakır et al. 2009; Przetakiewicz 2015), from which pathotypes 1, 2, 6, 8 and 18 are considered the most important forms of the fungus (Stachewicz 2002). Pathotype 18 is also considered to be among the most aggressive pathotypes, and only a few resistance genes have been characterized thus far.

Two dominant resistance genes, *Sen1* and *Sen1*–*4*, have been identified in diploid potato mapping populations. *Sen1* is located on potato chromosome 11 and confers resistance to *S. endobioticum* pathotype 1 (Hehl et al. 1999). In addition, according to Hehl et al. (1999), *Sen1* is closely linked to resistance gene-like sequences that are homologous to the *N* gene, which is responsible for TMV resistance in tobacco and which later was termed Xla-TNL (Bakker et al. 2011). Brugmans et al. (2006) identified *Sen1*–*4*, which is responsible for resistance to pathotype 1, on chromosome 4.

In two half-sib families, Ballvora et al. (2011) identified three Simple Sequence Repeat (SSR) markers that were linked to genes, which are responsible for resistance to *S. endobioticum* and mapped on chromosomes 1, 9 and 11: *Sen/2/6/18*-*I* on chromosome 1 confers resistance to pathotypes 2, 6 and 18; *Sen18*-*IX* on chromosome 9 confers resistance to pathotype 18; and *Sen1*-*XI* on chromosome 11 confers resistance to *S. endobioticum* pathotype 1. The resistance to pathotypes 2, 6 and 18 is highly correlated but independent of the resistance to pathotype 1. The *Sen* alleles that increased or decreased resistance to potato wart were inherited from both the resistant and the susceptible parents (Ballvora et al. 2011). Furthermore, Groth et al. (2013) detected a major Quantitative Trait Locus (QTL) responsible for resistance to pathotype 1 near *Sen1* on chromosome 11. QTLs responsible for resistance to pathotypes 1, 2, 6 and 18 have been detected on potato chromosomes 6, 8 and 11, and QTLs responsible for resistance to pathotypes 2, 6 and 18 have been detected on chromosomes 7 and 10. A QTL for resistance to pathotypes 6 and 18 has been detected on chromosome 2, and an additional QTL responsible for resistance to pathotype 2 was mapped on chromosome 1. Additionally, Obidiegwu et al. (2015) used the 8.3 k SolCAP SNP array to genotype a tetraploid potato population and identified new and previously known loci responsible for resistance to various *S. endobioticum* pathotypes on chromosomes 1, 3, 4, 10, 11 and 12. The Xla-TNL locus on chromosome 11 carried a major resistance locus, and several minor resistance loci were observed on various other chromosomes.

Several linkage maps have been constructed in potato (Bonierbale et al. 1988; Gebhardt et al. 1989; Jacobs et al. 1995; Van Os et al. 2006). Most potato linkage maps are based on diploid potato populations to facilitate genetic segregation and inheritance models in the tetraploid species (Bonierbale et al. 1988; Jacobs et al. 1995; Van Os et al. 2006; Felcher et al. 2012). Primary dihaploid lines derived from anther culture have been successfully used to develop markers for resistance against potato virus Y (Song et al. 2005), and a parthenogenic approach has been used to map nematode resistance (Pineda et al. 1993). However, the population sizes in both studies were very small with only 57 and 37 dihaploid individuals.

In this study, we analyzed a large dihaploid population consisting of 215 genotypes, and subsets of these genotypes were screened for resistance to *S. endobioticum* pathotypes 18 and 6, which are among the most significant pathotypes responsible for potato wart. The dihaploid individuals were genotyped using the 12.8 k SolCAP SNP array, and the marker data were used to identify loci responsible for resistance to potato wart. Additional molecular markers were developed to fine map the major resistance locus on chromosome 11, and the resistance locus was narrowed to approximately 780 kbp in the Xla-TNL region containing also *Sen1*. This fine mapping is a significant improvement in the resolution of genes responsible for resistance to pathotypes 18 and 6 at the Xla-TNL locus. The tightly linked markers were tested in tetraploid varieties and showed potential diagnostic value in different genetic backgrounds.

## Materials and methods

In order to provide an overview about the different plant materials, markers and mapping methods used, a scheme of the experimental approach is provided in Figure S1.

### Plant material used for the generation of the dihaploid potato population

The tetraploid cultivar `Karolin´ (bred by: NORIKA Nordring-Kartoffelzucht-und Vermehrungs-GmbH, Sanitz, Germany), which is resistant to *S. endobioticum* pathotypes 1, 2, 6 and 18, was used to construct a dihaploid population using the so-called prickle pollination with dihaploid inducer clones, i.e., IVP101 and IVP35, of the diploid wild potato species *Solanum phureja* (Hougas and Peloquin 1957; Hutten et al. 1994). Altogether, 215 dihaploid genotypes were used for the resistance phenotyping, genotyping, and genetic mapping. In addition, 50 tetraploid potato cultivars were used to determine the diagnostic value of selected molecular markers developed in this study. The pollinations with the dihaploid inducers were performed in a greenhouse on emasculated `Karolin´ flowers (Bartkiewicz et al. 2018). The seeds of the cross of `Karolin´ and *S. phureja* IVP35 were preselected based on the occurrence of an embryo spot. The seeds derived from the crosses were surface sterilized by a 30-s incubation in 70% ethanol, 2-min incubation in 0.5% sodium hypochlorite + Tween 20, and three 5-min washing steps with sterile distilled water. The seeds were germinated in vitro on Murashige Skoog medium (Murashige and Skoog 1962) solidified using 8.4 g plant agar (Duchefa Biochemie B.V., Haarlem, The Netherlands) per liter and containing 3% sucrose. The emerging seedlings were cultivated at 23 °C under a 16 h light/8 h dark cycle with light intensities of approximately 60 µmol m^−2^ s^−1^.

### Ploidy determination

The putative dihaploid seedlings were visually selected based on the lack of anthocyanin pigmentation in the nodes of the in vitro seedlings. Subsequently, the ploidy of the selected seedlings was determined by performing flow cytometry using a CyFlow Ploidy Analyzer (Partec, Münster, Germany). The leaf tissue (~ 1 cm^2^) from the in vitro plantlets was chopped using razor blades in nuclei extraction buffer. The plant nuclei were stained with 4′,6-diamidino-2-phenylindole using the CyStain UV Precise P Kit (Partec, Münster, Germany). The analyses were performed according to the manufacturer’s protocol, and at least 1000 nuclei were counted per sample. Parental genotypes with known ploidy were used as standards for the diploid and tetraploid genotypes.

### Resistance phenotyping

Resistance to *S. endobioticum* pathotypes 18 and 6 (hereafter abbreviated as P18 and P6) was determined as previously described by Ballvora et al. (2011) according to a modified Glynne–Lemmerzahl test (Glynne 1925; Lemmerzahl 1930) where the tubers were not covered with a moist soil/peat mixture after the inoculation. The pathotypes P18 and P6 used for inoculation originated from infested areas in the south of Germany. P18 was isolated in Tannroda, Thuringia, and P6 in Olpe, North Rhine-Westphalia. Instead of the abbreviations P18 and P6 we used, the pathotypes were given an initial letter of the place of discovery and a progressive index number (T_1_ for P18 and O_1_ for P6) by Hey (1957). The propagation of the isolates was also carried out according to the modified Glynne–Lemmerzahl method, in which the warts can be harvested every 3–4 weeks and used for further inoculations. All tests were performed in the lab of the Julius Kühn-Institut in Kleinmachnow. For each tuber-bearing genotype, between five and 40 tubers were inoculated. The disease symptoms were scored from 1 (highly resistant) to 5 (highly susceptible). The mean scores were calculated according to *M  *= [*a *+* 2b *+* 3c *+* 4d *+* 5e*]/*n*, where *a*, *b*, *c*, *d* and *e* are the number of tubers scored 1 to 5, and *n* is the total number of scored tubers. For the qualitative resistance mapping, the genotypes were considered resistant at a mean resistance score lower than 2.49 and susceptible at a mean resistance score higher than 3.51. Additionally, only genotypes with at least five successfully inoculated and scored tubers were considered. Twenty-six genotypes with medium resistance scores ranging from 2.5 to 3.5 were excluded for the qualitative resistance mapping of P18 resistance, and accordingly 13 genotypes were excluded for the qualitative resistance mapping of P6 resistance.

### DNA extraction

The DNA was extracted using a DNeasy Plant Mini Kit (Qiagen, Hilden, Germany) according to the manufacturer’s protocol from approximately 30 mg of dried leaf tissue, which was homogenized using a TissueLyser II (Qiagen, Hilden, Germany). The DNA concentration was determined using a NanoDrop 2000 spectrophotometer (Thermo Fisher Scientific Inc., Waltham, Massachusetts, USA).

### SNP genotyping using the 12.8 k SolCAP potato SNP array and Kompetitive Allele Specific PCR assay

Using the 12.8 k SolCAP potato genotyping array, the 215 dihaploid genotypes and the parental genotypes were genotyped for 12,808 SNPs. The parental genotypes were genotyped with two repeats. Custom genotyping was performed by Neogene Genomics (Neogene Genomics, Lincoln, Nebraska, USA) and has been described previously in Bartkiewicz et al. (2018). The SNP array results were validated using the following Kompetitive Allele Specific PCR (KASP) markers of the SNP markers that were most significantly linked to potato wart resistance: solcap_snp_c2_33740, solcap_snp_c2_33712, solcap_snp_c1_4319, solcap_snp_c1_4322, solcap_snp_c2_6082, solcap_snp_c2_6287, solcap_snp_c1_2275, solcap_snp_c2_6309 and solcap_snp_c2_6285. The KASP primers were designed by LGC Genomics (LGC, Hoddesdon, UK) using a KASP by Design assay based on the context sequence information provided by the Solanaceae Coordinated Agricultural Project (http://solcap.msu.edu/data/potato_69011_map_context_DM_v3_superscaffolds.txt). PCR was performed using 50 ng of genomic DNA in 10 µl reaction volume on an ABI StepOnePlus instrument (Thermo Fisher Scientific, Waltham, Massachusetts, USA) according to the protocol provided by LGC genomics.

### Construction of pools for the bulked segregant analysis (BSA)

Three bulks were constructed based on the results of the disease resistance screening. Bulk1 comprised three highly resistant genotypes (mean resistance scores ≤ 1.5), bulk2 comprised five resistant genotypes (mean resistance scores between 1.6 and 1.7), and bulk3 comprised seven susceptible genotypes (mean resistance scores ≥ 4.2). The markers showing banding patterns specific to the highly resistant and resistant bulks were tested in the individual genotypes of the bulks and the entire dihaploid population.

### Simple sequence repeat markers

Simple sequence repeat (SSR) markers were developed for the *Sen1* region on potato chromosome 11 using the SSRLocatorI software (Da Maia et al. 2008). The markers were PCR-amplified from 40 ng of genomic DNA using the primers listed in Supplementary Table S2. The forward primers were M13-tailed (5′-GTAAAACGACGGCCAGT-3′) at the 5′-end, and a second M13-forward primer labeled with IRD700 (Eurofins MWG, Ebersberg, Germany) was used (Schuelke 2000). The PCR mixes with a total volume of 20 µl contained 0.125 µM of the IRD700-labeled M13-forward primer, 0.025 µM of the marker-specific forward primer, 0.25 µM of the marker-specific reverse primer, 1 unit of DCS *Taq* polymerase (DNA Cloning Service e.K., Hamburg, Germany), 1 × Williams buffer (100 mM Tris–HCl (pH 8.0), 500 mM KCl, 20 mM MgCl_2_, and 0.01% gelatine) and 0.15 mM of each dNTP. The PCR conditions were the same as those described by Omondi et al. (2017). After performing the PCR, 100–250 µl of formamide loading dye (98% formamide, 10 mM EDTA, and 0.05% pararosaniline) was added, and the samples were denatured for 3 min at 95 °C. For each sample, 0.3 µl of diluted PCR product was size-separated on 6% polyacrylamide gels (Sequagel XR, National Diagnostics, Nottingham, UK) on a LI-COR DNA Analyzer 4300 (LI-COR, Lincoln, Nebraska, USA) according to the manufacturer’s protocol.

### Single-strand conformation polymorphism markers

Single-strand conformation polymorphism (SSCP) markers were developed for the *Sen1* region on potato chromosome 11 and PCR-amplified from 40 ng of genomic DNA using the primers listed in Supplementary Table S2. The PCR mixes were the same as those described above for the SSR markers. The PCR conditions were as follows: initial denaturation for 5 min at 94 °C; 30 cycles of 45 s at 94 °C, 1 min at 63 °C and 1 min at 72 °C; ten cycles of 30 s at 94 °C, 45 s at 52 °C and 1 min at 72 °C; and a final extension of 10 min at 72 °C. After performing the PCR, an equal amount of SSCP dye (95% formamide, 0.01 M NaOH, 0.05% xylene cyanol, and 0.05% bromophenol blue) was added, and the samples were denatured for 3 min at 95 °C. For each sample, 1 µl of the diluted PCR product was size-separated on 0.5 × MDE gels (0.5 × MDE ^®^ gel solution (Lonza Group Ltd., Basel, Switzerland), 0.6 × long run TBE buffer (80.4 mM Tris, 7.5 mM boric acid, and 1.5 mM EDTA), 5% glycerin, 0.05% APS, and 10 µl TEMED. The IRD-labeled single strands were detected using the Odyssey^®^ Infrared Imaging System (LI-COR, Lincoln, Nebraska, USA).

### Y1delATT-marker

The Y1delATT-marker developed by Obidiegwu et al. (2015) was PCR-amplified from 20 ng of genomic DNA using PCR mixes with a total volume of 25 µl contained 0.5 µM of each primer, 6% dimethyl sulfoxide (Roth GmbH, Karlsruhe, Germany), 0.4 M Betaine (Sigma-Aldrich, St. Louis, Missouri, USA), 1 unit of Bioline *Taq* polymerase (Bioline, Luckenwalde, Germany), 1 × Williams buffer (100 mM Tris–HCl (pH 8.0), 500 mM KCl, 20 mM MgCl_2_, and 0.01% gelatine) and 0.16 mM of each dNTP. The PCR conditions were the same as those described by Obidiegwu et al. (2015). The PCR products were detected by agarose gel electrophoresis.

### RNA isolation

For the extraction of RNA from the three resistant dihaploid genotypes, i.e., B35B-1, B35A-7 and B35F-6, and three susceptible dihaploid genotypes, i.e., B35C-8, B35F-10 and K12-3, 30–50 mg leaf tissue was frozen in liquid nitrogen and homogenized using a TissueLyser II (Qiagen, Hilden, Germany). The RNA extraction was performed using an RNeasy Plant Mini Kit (Qiagen, Hilden, Germany) according to the manufacturer’s protocol. Contaminating DNA was removed from the extracted RNA using a DNA-free™ Kit (Ambion, Thermo Fisher Scientific, Waltham, Massachusetts, USA) according to the manufacturer’s protocol.

### RNA-Seq data analysis

Ten micrograms of the total RNA from each of three resistant and three susceptible dihaploid genotypes was sent on dry ice to GATC Biotech (GATC Biotech AG, Konstanz, Germany), where cDNA library preparations were performed and sequenced on the Illumina platform using the 2 × 125 bp paired end mode. Transcriptome sequences specific to the resistant genotypes were identified using the following approaches:

1. Reads from resistant and susceptible genotypes were assembled and mapped against known Solanaceae resistance gene analogs (RGAs)
2. Using transcripts for RGAs specifically identified in the Xla-TNL region on potato chromosome 11 and
3. Other transcripts (from non NBS–LRR genes) specific to the resistant dihaploid genotypes were identified. Then, the identified contigs were used for the marker development.

For all approaches, assembly and mapping of reads to Solanaceae RGAs or the reference sequence of the Xla-TNL region was conducted with CLC Genomics Workbench 7.5 (Qiagen Bioinformatics, Denmark). For mapping, a similarity fraction of 95% was used as a threshold value. PCR primers were generated for transcripts with indels or SNPs between the resistant and susceptible genotypes in placing the 3´ region of one of the primers with three to five bases into the polymorphic region of the transcripts from the resistant genotypes.

### PCR markers derived from the RNA-Seq analysis

The PCR markers derived from the RNA-Seq analysis were amplified from 20 ng of genomic DNA using specific primers for three resistant-specific contigs, i.e., Kc8103 (forward primer 5′-GGGAAGTGCATGATTCAGAGC-3′, reverse primer 5′-GGCAGTTCCGTTATCCTAGTG-3′), Kc49 (forward primer 5′-TTGCTTTGTTTTCCCTCCGG-3′, reverse primer 5′-CATCAACTGGCTTCATTGGA-3′) and Kc19 (forward primer 5′-GTTCACTGTTTCATTTATGGACTGA-3′, reverse primer 5′-TTCAATTTTCCCCGGATCTT-3′), in a total volume of 25 µl. The reactions were performed according to the manufacturer’s protocol using MyTaq™ DNA polymerase (Bioline, Luckenwalde, Germany). The PCR products were detected by agarose gel electrophoresis.

### Marker-trait associations

The marker-trait associations were determined using R software version 3.1.3 (R development core team 2011) by performing a nonparametric Kruskal–Wallis rank test. The mean values of the resistance phenotyping and the genotyping results of the SNP array were utilized. The PCR marker data were transformed into a 1/0 matrix representing the presence (1) and absence (0) of the respective marker band. After a false discovery rate (FDR) adjustment which was conducted to minimize the number of false positive markers, a *p* value of 0.05 was chosen as the significance threshold indicating that a marker is significantly linked to resistance to *S. endobioticum* pathotypes 18 and 6.

### Genetic linkage mapping

The genetic mapping of the `Karolin´ population was performed using only single dose SNP markers indicated by a 1:1 segregation. Skewed markers or markers with missing values for more than 15 genotypes were not considered. To construct the linkage maps, LOD scores between 6 and 15 were chosen. For each potato chromosome, two to four linkage maps were constructed. A linkage analysis was performed in JoinMap^®^4 (Van Ooijen 2006) using the mapping function of Haldane (1919) and the regression mapping algorithm (Stam 1993). To fine map the resistance locus, SSR-, SSCP- and PCR marker data were included. The same mapping parameters were used as described for the genetic linkage mapping.

## Results

### Phenotypic analysis of wart resistance

**Fig. 1:**
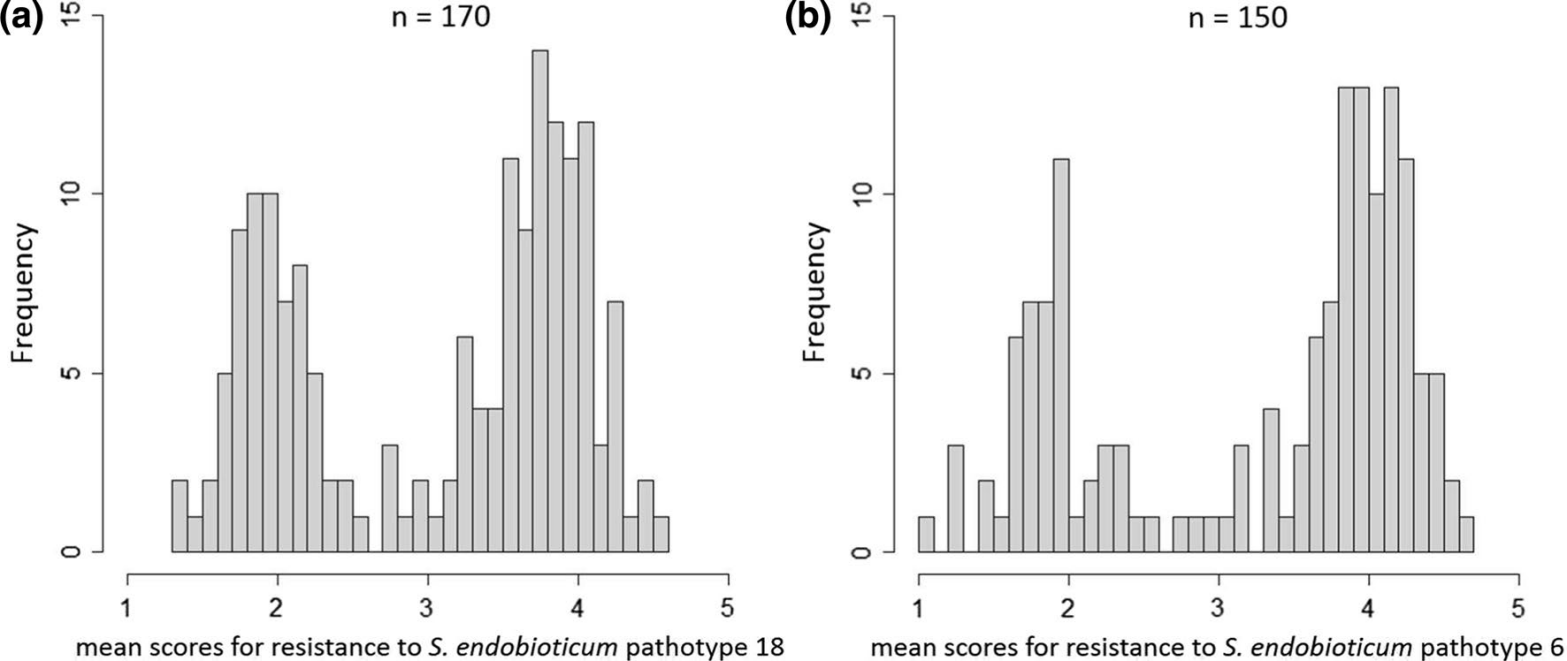
Distribution of the mean scores of the resistance to *S. endobioticum* P18 (**a**) and P6 (**b**). Altogether, 170 and 150 genotypes were successfully inoculated with P18 and P6, respectively

**Fig. 2:**
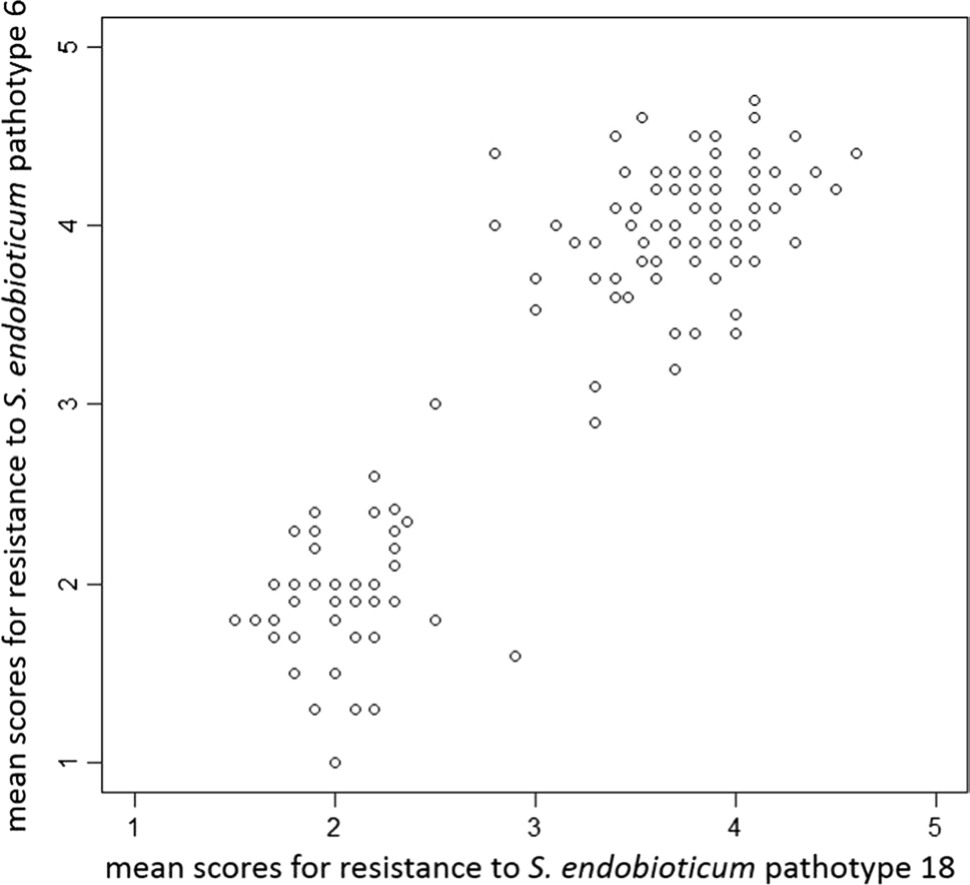
Correlation of the resistance to *S. endobioticum* P18 and P6 calculated according to Spearman. Mean resistance scores of 145 genotypes, for which phenotypic resistance data were available for both pathotypes, were plotted. Resistance to both pathotypes was highly correlated with a correlation coefficient of *r* = 0.8 (*p* value < 2.2e − 16)

Of the 215 dihaploid genotypes, 170 genotypes had a sufficient number of tubers and were successfully inoculated with P18, with five to 38 tubers each and mean resistance scores ranging from 1.3 to 4.6 (Fig. 1a). For 150 genotypes, five to 30 tubers were also successfully inoculated with P6 with mean resistance scores ranging from 1.0 to 4.7 (Fig. 1b). The distribution pattern of the mean resistance scores for both pathotypes displayed a clear bimodal distribution and resistances to both pathotypes were highly correlated in the individual genotypes (Fig. 2) with a correlation coefficient of *r* = 0.8 (*p* value < 2.2e − 16).

### Mapping of wart resistance loci

The mapping of the resistance to the two wart pathotypes was conducted using the following two strategies (Figure S1): first, a nonparametric mapping based on the quantitative wart scores and Kruskal–Wallis tests, and second, a conventional qualitative mapping approach was used in which the resistance scores were recoded as resistant (mean scores less than 2.49) or susceptible (mean scores higher than 3.51).

### Quantitative analysis of marker-trait associations

The phenotypic resistance scores were used to calculate the marker-trait associations. The SNP genotyping using the 12.8 k SolCAP SNP array resulted in 4679 segregating SNP markers within the population. These markers were used to perform Kruskal–Wallis tests to determine the association of resistance with P18 and P6. Ninety-nine SNP markers were identified that showed significant association with resistance to P18, including 82 markers from potato chromosome 11.

Of these markers, the 20 markers with the lowest *p* values (*p* ≤ 5E − 5 after FDR adjustment), which are most significantly linked to resistance to P18, were all located within a 5.6 Mbp region on the distal end of chromosome 11 (939,591 bp-3,080,240 bp, Table S3). Sixteen markers were located on chromosome 10 (Table 1). For one marker, no physical position could be identified in the potato genome browser. Eighty-seven significant markers were identified for P6, all of which were located on chromosome 11 (Table 1). The 14 markers with the lowest *p* values (*p* ≤ 5E − 5 after FDR adjustment) were all located at the distal end on chromosome 11 (939,591 bp-3,149,876 bp, Table S4), which is consistent with the results observed for P18. Altogether, 76 markers were identical to those identified to be significantly linked to the resistance to P18. Information regarding the individual SNP markers that were significantly linked to P18 and P6 is provided in Supplementary Tables S2 and S3.

**Table 1:** Number of SNP markers significantly linked to the resistance to *S. endobioticum* P18 and P6

Chromosome	Physical region (Mbp)	Number of P18 markers	Number of P6 markers	Identical markers for both pathotypes
11	0–5.6	40	34	34
	6.2–9.6	12	13	12
	10.0–14.3	21	20	20
	20.8–28.0	5	6	5
	30.5–41.8	4	13	4
10	55.7–58.1	16	–	–

The number of markers is listed for both pathotypes in the respective physical regions on chromosomes 10 and 11 according to the Spud DB Genome Browser PGSC v4.03. The number of identical markers for both pathotypes is also listed

For nine of the markers that were most significantly linked to resistance to both P18 and P6, a KASP assay was performed to validate the SNP marker data. Both methods showed high consistency regarding the genotyping, and only one marker, i.e., solcap_snp_c2_6082, differed for two dihaploid genotypes (Table S5).

### Fine mapping of qualitative wart resistance

A genetic linkage map was generated using 2548 single dose SNP markers displaying a 1:1 segregation. Altogether, 45 linkage groups were obtained (Bartkiewicz et al. 2018). The nine markers that were most significantly associated with the resistance to *S. endobioticum* P18 and P6 (Tables S2, S3) were located in a single linkage group.

For the qualitative resistance screening, the genotypes were classified as either resistant (mean resistance score ≤ 2.49) or susceptible (mean resistance score ≥ 3.51). After excluding 26 and 13 individuals with medium resistance scores ranging from 2.5 to 3.5, 144 and 137 genotypes were clearly classified as resistant or susceptible to P18 and P6, respectively. Of these genotypes, 61 genotypes were resistant and 83 genotypes were susceptible to P18, corresponding to a 1:1 segregation ratio for P18 (χ^2^ = 3.361; *p* > 0.05). The segregation of resistance scores for P6 was skewed toward a 1:2 ratio (χ^2^ = 0.179; *p* > 0.05) with 48 resistant and 89 susceptible genotypes.

The mapping of the qualitative resistance as a binary marker allowed the positioning of the resistance locus for P18 on chromosome 11 between the two SNP marker groups solcap_snp_c2_33740/solcap_snp_c2_33712 and solcap_snp_c1_4322/solcap_snp_c1_4319, spanning a physical distance of 1.15 Mbp. The recombinant genotypes for these two marker groups are listed in Table 2. The qualitative mapping of the resistance to P6 placed the resistance locus in the same interval.

**Table 2:** Molecular markers used to fine map the major locus responsible for the resistance to *S. endobioticum* P18 on chromosome 11 along with their physical position and the number of recombinant genotypes for each marker

Marker	Marker type	Physical position (bp)	Recombinant genotypes
solcap_snp_c1_4322/solcap_snp_c1_4319	SNP	939,581	K8-1
SSCP4348	SSCP	1,163,786	None
Kc8103	PCR	1,407,791	None
RK7	SSR	1,610,809	None
RK75	SSR	1,630,787	None
RK76	SSR	1,637,061	None
RK70	SSR	1,665,423	None
RK69	SSR	1,667,558	None
RK91	SSR	1,683,357	None
RK36	SSR	1,716,722	K14-3
SSCP13	SSCP	1,768,997	K14-3
SSCP14	SSCP	1,771,582	K14-3
SSCP15	SSCP	1,776,834	K14-3
Y1delATT	PCR	1,844,035	K14-3; B35H-4
solcap_snp_c2_33740/solcap_snp_c2_33712	SNP	2,089,292	K14-3; B35H-4; B35G-8

**Fig. 3:**
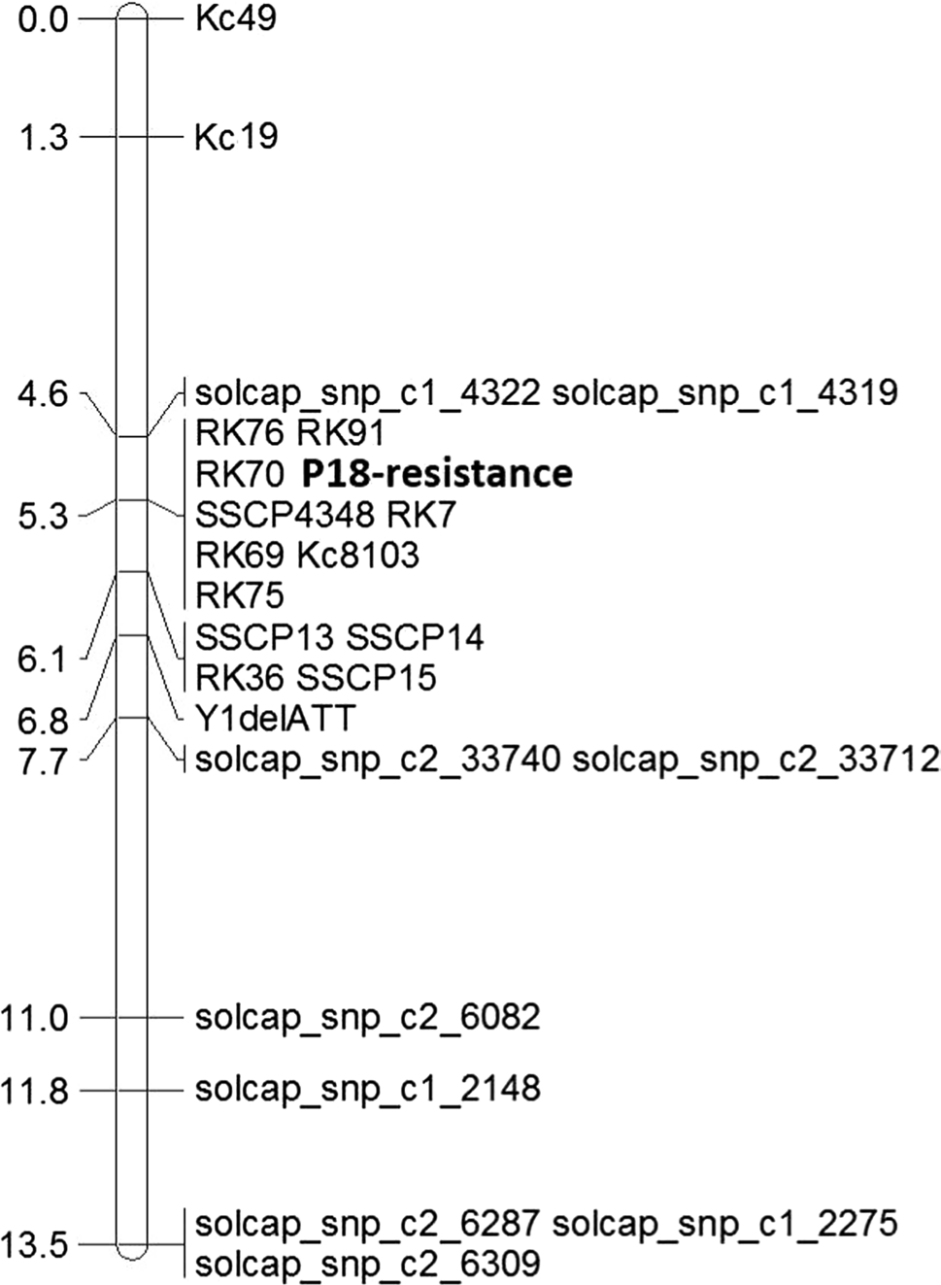
Local genetic map of the locus responsible for the resistance to *S. endobioticum* P18 on chromosome 11. Eight markers showed no recombinant genotypes to the resistance locus (P18-resistance) at 5.3 cm, while four markers were recombinant for one genotype at 6.1 cm. For the Y1delATT marker, two genotypes were recombinant

To further narrow the marker interval around the resistance locus, 51 SSR and 53 SSCP markers were developed for the 1.15 Mbp genomic region (Table S2). The comparison of the RNA-Seq data between the resistant and susceptible dihaploid genotypes allowed the identification of contigs specific for the resistant dihaploid clones. From these, markers were developed that allowed the amplification of polymorphic DNA fragments specific for the resistant genotypes. Additionally, the Y1delATT marker from previous publications (Obidiegwu et al. 2015) was used for the fine mapping. The markers were tested in a “bulked segregant” analysis using three DNA pools consisting of genomic DNA of highly resistant, resistant and susceptible genotypes. The entire dihaploid population was screened for marker bands specific to the highly resistant and resistant pools. Altogether, seven SSR markers, four SSCP markers, three PCR markers and the Y1delATT marker were used to fine map the resistant locus (Fig. 3). The approximate physical positions of these markers based on the reference genome and the number of recombinant genotypes are listed in Table 2. Six SSR markers, one SSCP marker and one PCR marker showed no recombination to P18 and P6 resistance. SNP markers solcap_snp_c1_4322/solcap_snp_c1_4319 and SSR marker RK36 flank the resistance locus on each side with one recombinant genotype.

By mapping the additionally developed molecular markers onto the reference genome, the genomic region around the resistance locus was narrowed from approximately 1.15 Mbp to approximately 777 kbp.

**Fig. 4:**
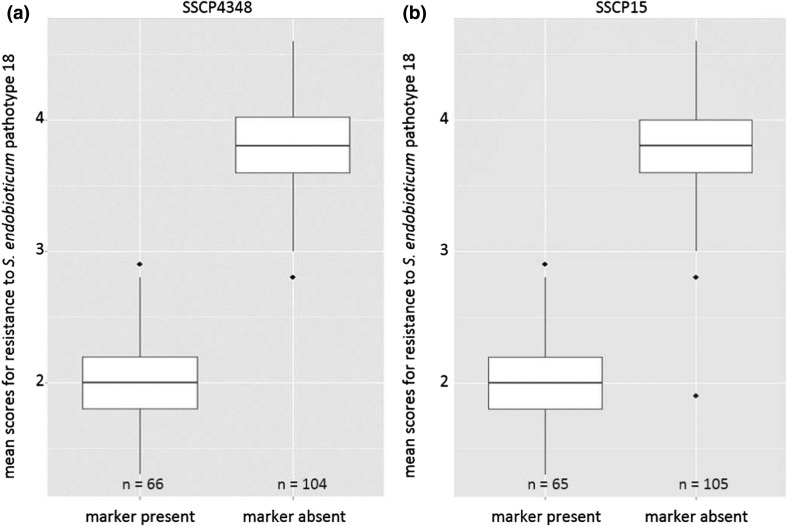
Boxplots for the distribution of the mean scores of resistance to *S. endobioticum* P18 for markers SSCP4348 (**a**) and SSCP15 (**b**) in dependence of the presence and absence of the markers in the 170 dihaploid genotypes of the population. The boxes represent the 25th and 75th quartiles, and the medians are indicated by the bold line

The phenotypic effects of these markers were calculated in the entire dihaploid population, including the genotypes with medium resistance scores for P18 that were previously excluded (Table S6). The mean scores of the group with the markers ranged from 2.01 to 2.08, and the mean scores of the group without the markers ranged from 3.77 to 3.80. The phenotypic distribution of the resistance to P18 for markers showing no recombinant genotypes and markers showing one recombinant genotype, K14-3, is shown in Fig. 4. The phenotypic resistance scores ranged from 1.3 to 2.9 in the group with a present marker and 2.8 to 4.6 in the group without the marker for the individual genotypes. The recombinant genotype K14-3 (Fig. 3, Table 2) had a mean resistance score of 1.9.

### Diagnostic value of the developed markers

To test the diagnostic value of the newly developed molecular markers, selected markers were also tested in 38 tetraploid potato varieties with available phenotypic resistance scores for P18 (Table 3). The best results were obtained with markers Kc8103 and RK36, each of which identified four non-matching varieties. `Jutrzenka´ and `Saphir´ were both classified as resistant to P18 but showed absent genotypes for both markers, which is a false negative rate of 25%. In contrast, the varieties `Merano´ and `Milek´ were susceptible to P18 but showed a present genotype with both markers, which equals a false positive rate of only 6% for the markers (Figure S2). Forty-five varieties with known resistances for P6 were tested with the two markers, and six non-matching genotypes were identified (Table 3). For P6, the markers had a false negative rate of 18% (two out of eleven resistant varieties with missing marker fragments) and a false positive rate of 11.7% (four of the 34 susceptible varieties showed a fragment). The other tested markers were either not polymorphic in the tetraploid varieties or did not show a clear segregation pattern for resistant and susceptible varieties.

**Table 3:** Analysis of the diagnostic markers Kc8103 and RK36 in tetraploid potato varieties with known resistances to *S. endobioticum* P18

Variety	Resistance classification P18	Resistance classification P6	Genotypes for markers Kc8103 and RK36
`Agria´	NA	S2	Absent
`Alegria´	S2	S2	Absent
`Altus´	S1/S2	S1/S2	Absent
`Avano´	S1	S1	Absent
`Axion´	NA	S1/S2	Absent
`Birte´	S2	S2	Absent
`Burana´	S2	S2	Absent
`Campina´	S2	S1	Absent
`Combi´	S1/S2	S1/S2	Absent
`Concordia´	S2	S2	Absent
`Cumbica´	S2	S2	Absent
`Deodara´	S1/S2	S1/S2	Absent
`Desirée´	S1/S2	S1	Absent
`Django´	NA	S1	Present
`Eurobravo´	NA	S1	Absent
`Finka´	S2	S2	Absent
`Gandawa´	NA	R1/R2	Present
`Gawin´	R1/R2	R1/R2	Present
`Heidi´	S2	S2	Absent
`Ibis´	NA	S1	Present
`Igor´	R1	R1	Present
`Ikar´	R1/R2	R1	Present
`Jasia´	S1	R1/R2	Absent
`Jutrzenka´	R1/R2	NA	Absent
`Kuba´	NA	R1/R2	Present
`Laura´	S2	S2	Absent
`Lilly´	S2	S2	Absent
`Marabel´	NA	S2	Absent
`Megusta´	R2	R2	Present
`Merano´	S1/S2	S1	Present
`Milek´	S1/S2	NA	Present
`Miriam´	S1/S2	NA	Absent
`Natascha´	S2	S2	Absent
`Opal´	S2	S2	Absent
`Panda´	S1	NA	Absent
`Pasat´	NA	S1/S2	Absent
`Pasja´	NA	S1/S2	Present
`Renate´	NA	S1	Absent
`Romanze´	S2	NA	Absent
`Rudawa´	NA	S1	Present
`Saphir´	R1	R1/R2	Absent
`Seresta´	S1	NA	Absent
`Sleza´	R2	R1/R2	Present
`Soraya´	S2	S2	Absent
`Talent´	S1/S2	S1/S2	Absent
`Toccata´	S2	S1	Absent
`Tomensa´	S1/S2	S1/S2	Absent
`Troja´	S2	S1	Absent
`Ulme´	R1/R2	R1/R2	Present
`Venezia´	S2	S2	Absent
`Zagloba´	NA	R1/R2	Present

R1 indicates highly resistant and resistant varieties, R2 indicates weakly resistant varieties, S1 indicates slightly susceptible varieties, and S2 indicates highly susceptible varieties. For P18, eight varieties were classified as resistant (R1 or R2), and 30 varieties were classified as susceptible (S1 or S2). For P6, eleven varieties were classified as resistant (R1 or R2), and 34 varieties were classified as susceptible (S1 or S2). Cultivars for which resistance data for either of the pathotypes were not available are marked with “NA”

## Discussion

In this study, we present molecular markers that tag a major resistance locus on potato chromosome 11 with no recombinant genotypes observed in our dihaploid potato population. To our knowledge, this study is the first to narrow the position of the resistance locus in the Xla-TNL region for P18 and P6 to less than 800 kbp, establishing the basis for further genetic analyses of wart resistance and the identification of the underlying resistance gene or genes.

The distributions of the phenotypic resistance scores were bimodal for both pathotypes (Fig. 1) and showed a 1:1 segregation ratio for P18 after excluding a few genotypes with medium resistance scores between 2.49 and 3.51, indicating that one major resistance gene is responsible for the resistance. The phenotypic distribution of resistance segregating as a monogenic character has been described in earlier studies for pathotype 1 (Hehl et al. 1999; Brugmans et al. 2006; Obidiegwu et al. 2015). The bimodal phenotypic distribution of resistance to pathotypes 2, 6 and 18 has also been observed by Obidiegwu et al. (2015); however, the distribution did not fit a 1:1 segregation ratio in their study, where for the SNP genotyping, 79 genotypes with intermediate resistance scores were excluded, and the resistance mapping was performed only with 54 selected genotypes. Using the resistant parent used in the study conducted by Obidiegwu et al. (2015), we observed a clearer segregation of resistance, indicating the presence of a major dominant factor responsible for the resistance segregation in our population. The difference from the tetraploid population used by Obidiegwu et al. (2015) could be explained by additional alleles contributed by the second tetraploid parent in their study that could have modified the resistance in their tetraploid progeny. Furthermore, the ploidy level could account for the variation resulting in genotypes with intermediate resistance scores because loci displaying dose-dependent effects lead to a broader variation among tetraploid progeny than in dihaploids. In our study, we excluded 26 medium scored genotypes to avoid false positives in the classification of the genotypes as resistant. Nevertheless, the developed markers were tested in all genotypes and showed the expected results, even in the medium scored genotypes. The segregation of the resistance scores for P6 was slightly skewed toward susceptibility and did not correspond to a 1:1 segregation. This skewed distribution for P6 resistance could be easily explained by the fact that certain genotypes resistant to P18 did not produce enough tubers to be tested with P6. Because the resistance scores for both pathotypes were highly correlated (Fig. 2), these genotypes could also be resistant to P6 with a high probability, resulting in a 1:1 segregation for P6. The high correlations between the resistances to pathotypes 2, 6 and 18 have been previously described (Ballvora et al. 2011; Groth et al. 2013).

Using the SNP genotyping data, the qualitative resistance mapping of the clearly classified genotypes identified the resistance loci for both pathotypes on potato chromosome 11 (Fig. 3) in the Xla-TNL region, which is known as a major locus responsible for resistance to potato wart pathotype 1 (Hehl et al. 1999; Gebhardt et al. 2006; Ballvora et al. 2011, Groth et al. 2013) and pathotypes 2, 6 and 18 (Obidiegwu et al. 2015). Of the 82 markers that were significantly linked to the resistance to P18 located on chromosome 11, 92.7% (76 markers) were also significantly linked to the resistance to P6 (Table S3, S4). In addition, 16 markers identified on chromosome 10 were significantly linked to the resistance to P18. For P6, eleven additional markers identified on chromosome 11 were not identified to be significantly linked to the resistance to P18. The resistance locus for P18 on chromosome 10 is consistent with the resistance locus identified by Groth et al. (2013), indicating that the resistance to P18 is controlled by a second minor resistance locus on chromosome 10 in addition to the major resistance locus on chromosome 11, which is not the case for P6. A KASP assay of the nine most significantly linked SNP markers confirmed the genotyping results of the SNP array (Table S5). Only one marker showed different genotyping results for two genotypes. However, one genotype was excluded from the resistance analyses because only three and two tubers were successfully inoculated with P18 and P6, respectively. The other genotype was excluded from the resistance analysis of P18 because it showed a medium resistance score of 3.3.

The development of additional SSR, SSCP, and PCR markers allowed for the fine mapping of the major resistance locus on chromosome 11 by narrowing the locus from approximately 1.15 Mbp to approximately 777 kbp. The physical distances of markers showing one and zero recombinant genotypes are 33,365 bp between markers RK91 (no recombinant genotype) and RK36 (K14-3 as a recombinant genotype) and considerably higher between solcap_snp_c1_4322/solcap_snp_c1_4319 (K8-1 as a recombinant genotype) and SSCP4348 (no recombinant genotype) with 224,205 bp, indicating that there are possibly additional markers in this region to further narrow down the resistance locus which stayed undetected in this study. Altogether, the developed markers in this study allowed a clear distinction between resistant and susceptible genotypes in our dihaploid population (Table S6; Fig. 4).

Although wart resistance is highly dependent on the genetic background of the respective varieties (Khiutti et al. 2012), we determined the diagnostic value of our developed makers by screening 38 tetraploid potato varieties with known resistance to P18 and 45 varieties with known resistance to P6. The markers Kc8103 and RK36 showed the most promising results. Of the 38 tested varieties for P18, four non-matching genotypes were observed, and the markers were diagnostic in 89.5% of the cases (Table 3; Figure S2). Of the 45 tested varieties for P6, the markers were diagnostic in 86.6% of the cases with six non-matching genotypes (Table 3). To the best of our knowledge, these two markers developed in our study are the first markers to show potential diagnostic value for resistance to P18 and P6. Thus far, only one marker, i.e., Nl25, has been reported to show high linkage to the Xla-TNL locus carrying *Sen1* and, therefore, resistance to pathotype 1 (Gebhardt et al. 2006). Within the CORNET project SynTest (establishment of a harmonized methodology for testing the resistance of potato cultivars to potato wart disease in the EU), the usability of three DNA markers (Nl25, GP125 and Stl046) was tested to evaluate the resistance to pathotype 1. With seven non-matching varieties out of 89 tested, the marker Nl25 was diagnostic in 92% of the cases (K. Flath, personal communication). Unfortunately, reliable phenotypic resistance data for P18 and P6 are not available for more tetraploid varieties to further substantiate the diagnostic value of markers Kc8103 and RK36 in different genetic backgrounds. Nevertheless, our results indicate that the same resistance locus plays a role in the resistance reaction in different genetic backgrounds displayed by different potato varieties with additional resistance loci likely present on various other chromosomes.

## Conclusions

In this study, we analyzed resistance to potato wart P18 and P6 using a monoparental dihaploid population derived from a highly resistant tetraploid cultivar. The resistance to both pathotypes can be resolved into a major factor at the Xla-TNL locus on chromosome 11 of potato. The resistance to P18 is additionally influenced by minor QTLs on chromosome 10. By converting the resistance scores into qualitative scores, we fine mapped the resistance to P18 and P6 to a genomic interval of less than 800 kbp using several linked markers without recombination. This study provides the highest resolution in mapping the resistance to P18 and P6 thus far and opens opportunities for screening candidate genes in the future. Furthermore, several developed markers showed potential diagnostic value for resistance to *S. endobioticum* P18 and P6 in 38 and 45 tetraploid varieties with different genetic backgrounds. To the best of our knowledge, these markers are the first to possess diagnostic value for this pathotype. The use of DNA marker techniques will provide a cost-effective evaluation of P18 and P6 resistance. This will considerably speed up the breeding progress. Potato cultivars with improved resistance will open new markets in Eastern Europe and Russia and will enable a more efficient control of potato wart disease.

### Author contribution statement

B planned and performed the experiments, analyzed the data, prepared all tables and figures and wrote the manuscript. FC generated the dihaploid potato population and determined the ploidy of genotypes by flow cytometry. DT performed the RNA-Seq experiment and developed the PCR markers derived from the RNA-Seq analysis. JL, JS, ET and HH provided the plant material for the crossings and tuberized the dihaploid genotypes. KF performed the wart resistance phenotyping. ML planned and supervised the experiments and corrected the manuscript. TD planned and supervised the experiments and wrote part of and corrected the manuscript.

## Electronic supplementary material

Below is the link to the electronic supplementary material.

Supplementary material 1 (DOCX 382 kb)
