# Supplementary material for: Improved genetic resolution for linkage mapping of resistance to potato wart in monoparental dihaploids with potential diagnostic value in tetraploid potato varieties
Source: Theor Appl Genet. 2018 Aug 29;131(12):2555–66. doi: 10.1007/s00122-018-3172-9 (PMC6244520; doi:10.1007/s00122-018-3172-9)

Improved genetic resolution for linkage mapping of resistance to potato wart in monoparental dihaploids with potential diagnostic value in tetraploid potato varieties

Annette Bartkiewicz^1,6^, Friederike Chilla^1,5^, Diro Terefe-Ayana^1,2^, Jens Lübeck^3^, Josef Strahwald^3^, Eckhard Tacke^4^, Hans-Reinhard Hofferbert^4^, Kerstin Flath^5^, Marcus Linde^1^, Thomas Debener^1^*

^1^ Institute of Plant Genetics, Department of Molecular Plant Breeding, Leibniz University Hannover, Hannover, Germany

^2^ Westhoff, Südlohn-Oeding, Germany

^3^ SaKa Pflanzenzucht GmbH & Co. KG, Windeby, Germany

^4^ Böhm-Nordkartoffel Agrarproduktion GmbH & Co. OHG, Ebstorf, Germany

^5^ Julius Kühn-Institut, Kleinmachnow, Germany

^6^ DHD-Consulting GmbH, Hildesheim, Germany

* Correspondence:

Prof. Dr. Thomas Debener

[debener@genetik.uni-hannover.de](mailto:debener@genetik.uni-hannover.de)

**Table S1** List of the dihaploid genotypes used for the Bulked Segregant Analysis, including their mean resistance scores.

| Bulk | Genotype | Mean resistance score |
| --- | --- | --- |
| Highly resistant | B35B-1 | 1.3 |
| Highly resistant | B35A-7 | 1.4 |
| Highly resistant | B35F-6 | 1.5 |
| Resistant | K2-1 | 1.6 |
| Resistant | B35C-18 | 1.6 |
| Resistant | B35E-9 | 1.7 |
| Resistant | B35L-1 | 1.7 |
| Resistant | K22-5 | 1.7 |
| Susceptible | K28-1 | 4.2 |
| Susceptible | K7-1 | 4.2 |
| Susceptible | K8-1 | 4.2 |
| Susceptible | K41-3 | 4.3 |
| Susceptible | B35C-8 | 4.3 |
| Susceptible | B35F-10 | 4.3 |
| Susceptible | K12-3 | 4.4 |

**Table S2** List of primer pairs for the SSCP and SSR markers used for fine mapping of the resistance locus on potato chromosome 11. Primer sequences and the expected PCR product size are given. Forward primers were tailed with a universal M13 sequence (5’-GTAAAACGACGGCCAGT-3’) at the 5’-end for infrared labelling of the PCR fragments

| **marker name** | **marker type** | **forward primer (5‘ to 3‘)** | **reverse primer (5‘ to 3‘)** | **product size [bp]** |
| --- | --- | --- | --- | --- |
| SSCP4332 | SSCP | TGTAGCCACAATCACAGCGA | TTTCTTTGTCGCGGCAACTG | 187 |
| SSCP13493 | SSCP | AATGGGTGGACGCAGGAAAT | TCTGAGCTAACACTAGTCCCA | 207 |
| SSCP13512 | SSCP | TACCTCGGAACCAAGGGAGA | AGATTGTTCGATCTCTCAGGCA | 177 |
| SSCP13517 | SSCP | TCTAAGCCAAAGATCCGCCT | TGATCATGAACAGCTCGACACA | 116 |
| SSCP4346 | SSCP | GGCTTCGAGTCATCCTGGTT | TGTAAACATGGGGAGCTTCGT | 168 |
| SSCP4348 | SSCP | AAGAAACCTGCAGGGAGAGC | AGGTCTAGCATTCAGGGCCT | 149 |
| SSCP13533 | SSCP | ACAAAGGCCCTAAAACGCGA | TCTTCGCCCTGCAGAACATT | 177 |
| SSCP13540 | SSCP | GGCTTGTGAAATTGCCGGAG | GGCACAAGAAGCAACAGGC | 127 |
| SSCP13555 | SSCP | AAGACTGCGTGCTTTGCTTG | GTCTCAACACCAGTAGCCACA | 103 |
| SSCP13562 | SSCP | TGCTTAGCTGTGTGAGTACCG | AGAATGGATTCCGAGCTGGT | 224 |
| SSCP4355 | SSCP | CCTCAAAGTCAACGCATGCC | CTGGCCGGAAAACCTTTTCG | 112 |
| SSCP57494 | SSCP | TCGATTGTTATCCCGGCCAG | AACCCCTGCGTTATGATTCG | 112 |
| SSCP37168 | SSCP | GAGTTCTCCGGCAACTGGAA | TTCACCCGAAGCAGCTTCAT | 129 |
| SSCP37175 | SSCP | GCTGTGGGTGTTGCTTTTGT | CTGCACGTCGTTGGAGAGAT | 126 |
| SSCP37177 | SSCP | TGGAAGACTCAAGAGGGAAAACC | TCGCTCCAGTACTACAACTCA | 231 |
| SSCP37188 | SSCP | GAAAATGATCAAGCAACCACAGT | TGTCAGGCATATATTGTACCGAG | 153 |
| SSCP37199 | SSCP | ATCTCAACTTTCAGGGCGCT | GTCCTCCTCCGGTTGGTTC | 187 |
| SSCP11113 | SSCP | GAGGCTTCATCAACTTGGAGC | AGTGGCAATCAATTTTTCCACT | 163 |
| SSCP37218 | SSCP | GCCTGAAAATTGGAATGTGTCG | TCAGTATCACATTCTGAATCGTCG | 183 |
| SSCP37220 | SSCP | AGATCCCAAGTTGGTTCCACC | TGGATGTGCAAAGTACCTTCCT | 164 |
| SSCP37216 | SSCP | TGCCGAATGACATCTCTGCT | TGGTGTCAACTAGGTTGCCA | 198 |
| SSCP37225 | SSCP | TCCATTGTATGCACGCAGGT | TTTCGACTTCCACTCCGAGC | 168 |
| SSCP13481 | SSCP | GCTGGAGACGATGTTGAACC | AGGGTTTGGCTTGGAACGTT | 173 |
| SSCP13508 | SSCP | TCCAGGTTTCATTTCATTTCATGGATCA | TCGAGACACGACCCCATAGT | 146 |
| SSCP13479 | SSCP | GTCGTCATGAGAAAGAAATACACGA | TCTTCCATATGCGCGGAGAC | 264 |
| SSCP4337 | SSCP | GGCTGTCACTATACATGCGA | TCACCTGGACTTCAACGAGAC | 201 |
| SSCP13483 | SSCP | GACCTGCAAAATATTTCTAAAAGAAGG | GGTCCTGGAGTTACCTGTCG | 150 |
| SSCP13496 | SSCP | GTAGTGCCTTGGAGGTTGCT | TGTAGACCCGTTGTTTGGCA | 129 |
| SSCP13505 | SSCP | TGCACGCTTCAAACTTCTTACC | TCTTCAGAAACCGCACAAGGA | 200 |
| SSCP13509 | SSCP | AGCTTTACTTGATGCCCGCT | CGCAAGTTTCTCAGATGTGACA | 217 |
| SSCP13510 | SSCP | TCGAGATATTCTAAATACGGGAAACT | TGAAATTTCATTTGGGCTGACTT | 210 |
| SSCP13527 | SSCP | TGCTGACAATTTTCGAAACACA | GCAAGTCACTAAGAGAATGAGCC | 181 |
| SSCP37230 | SSCP | AGGCCAATTTTCAGTCTCAACA | ACAGATGTTCTTTAGTACACATTATGC | 190 |
| SSCP10 | SSCP | TCACTTATGTTATAATGTTGTCGGAGT | TGCTCCAAAGTAAACAAACTCAA | 192 |
| SSCP11 | SSCP | GAGCCAAGATGACAACAACGG | TTTTTCACAGGACGAATATCGATT | 100 |
| SSCP12 | SSCP | TCTGTATCATTTGTATATCTTGTTGCT | TTACCTTGGAAGCAGAGGGG | 190 |
| SSCP13 | SSCP | ATGAGCAAGAAATCTAAGTCTAGCA | CGCCAACATCACGACGTTC | 190 |
| SSCP14 | SSCP | CCTCATCATGGCTTCACTGGA | GTTTTGAGCTTGCCTTGGCA | 230 |
| SSCP15 | SSCP | CATCTGTGTCTGTTGGCACG | CGCTGCTATGTTTCCGCTTC | 235 |
| SSCP16 | SSCP | AGATTCAACAGTTGGATCAGGGAA | ACCACATACTCGACAAGCTGTC | 393 |
| SSCP17 | SSCP | GTCCAATTGTAAACATTATGAGCAGT | CCCTTAAGCCATGGGAGACA | 303 |
| SSCP18 | SSCP | TCTTGTGGGGTTTGAGGCAG | GCAGACACGGTGACCAAAAC | 354 |
| SSCP19 | SSCP | CAGCTCCAACTCCTCAAGCA | TCGCAGCTGAGGCATCTAAG | 165 |
| SSCP20 | SSCP | TGGTGTTGTACGATGCCTCC | GACATGGAAAGGGTGAAATGAGT | 275 |
| SSCP21 | SSCP | AAATATGTCCTCCACCGGCC | GCTCAAGAGAAAGAAGCGGC | 330 |
| SSCP22 | SSCP | ACGATGGATCTTGCCTCTCC | CCGACCTGTGGGAATACAATGA | 213 |
| SSCP23 | SSCP | AACAGGGTATGCAATGGCCT | CTTTTTCGATTGCTCCCGGC | 315 |
| SSCP24 | SSCP | GCAGGGGTTTTCATTGATAGATT | TGGAAGCTTGTTTGAACGCA | 307 |
| SSCP25 | SSCP | TCACTGCCTCTTCTTGTCCAC | CGACACCGTTTTCCATTCCTG | 284 |
| SSCP26 | SSCP | CAAGTCATTGAAGCGATCTGCC | GCCAGTGTGGATAATGCTGC | 204 |
| SSCP27 | SSCP | TGCAAAGGTGGCTTCATTTGA | GCCGGAAAGATTAACAGGTGTG | 288 |
| SSCP28 | SSCP | ACTCACCTGCAAAACCACTGA | GCACCATGAGATTGTTGTAGCC | 403 |
| SSCP29 | SSCP | CAAATACATCATGTTGCAATGTAGACA | ACGATCTTCAAATATTACAAAAGCAAC | 320 |
| RK83 | SSR | ATGCCATCATAGCCTTGTGA | TTCTCTCCCCCTCTCATTATCA | 174 |
| RK105 | SSR | ATTAACTATCACCGCGTGCC | CCTAGGTCACAACATCCCAAA | 110 |
| RK51 | SSR | AAACCTGCAGTTTCCTCTGG | CATGCCTTTACGTATATCGTCTTG | 255 |
| RK48 | SSR | TGATCGAGTTAGGTTGGTTTTG | TCGTGGTCATTAACAAATCGTC | 167 |
| RK44 | SSR | TGCTGACACAAAACGATTGA | AATCCCTTAAGCCATGGGAG | 181 |
| RK61 | SSR | ACCACCACCACTTGCTCC | AAGCAGCCAAAAATGTAGGG | 139 |
| RK115 | SSR | AACCTGCACCAGCTAGCACT | TTGGTAGAGTCTTGGAAAGCC | 211 |
| RK65 | SSR | AGGGCAACAATTTGTAACGG | CCTTACAAGGCTCAAGGCTG | 279 |
| RK57 | SSR | TTATAACCCAGGCCCAACAA | CCTGAATCGCACTGCTACAA | 188 |
| RK46 | SSR | AATTAATTCTAAGCAGCCGCC | TTTTTCCGATAGATTTGCCG | 141 |
| RK79 | SSR | CCCAGAGCTCACTTGTGAAA | TGAATGGGATTGGAAGAGGA | 264 |
| RK78 | SSR | TTCCGCAAGTTCGATGTATCT | CGGCCAACTTGTCAACTACA | 238 |
| RK95 | SSR | AGCTCAAGGGGTTCTGGAAT | CCTGACATTATGCTGGTGGA | 240 |
| RK77 | SSR | GGTCTATTGGGCTTTATGCG | AGCATTTTGATTCGCTTCGT | 270 |
| RK99 | SSR | TGTCTCTTTTGAGTTGCCCA | TCACCTCAAAGTGGAATTCAGA | 253 |
| RK2 | SSR | AAGGCATCTCTTTCCCACAA | GGAACGTCTTCTGAAATCGG | 225 |
| RK33 | SSR | CATCAAACCACCCTTCACCT | GTAAGGTGTGGGGATCATGG | 250 |
| RK80 | SSR | ATCTCTAGCTGCCCATCCAA | TTGTACAGACAAGCCATGACG | 241 |
| RK25 | SSR | CTTGTTCCCAACCAAACGAC | GGGTCGATGATCCGTTTATT | 279 |
| RK29 | SSR | TAACTCCAGGGACCCAAGAA | ATTGGACTTGCTGAACCTGG | 259 |
| RK22 | SSR | ATGAAGATTCAGCCAGCAAA | TGCTTCTTCTTGTCTTGCTTTC | 274 |
| RK13 | SSR | TCCATTGTTCTCCTTGTCCA | CCACCATTTGGATTTTGGAT | 247 |
| RK10 | SSR | CCCACCCCACCTCAATTAGT | AACACTGCCAGGTGGTTTTT | 234 |
| RK7 | SSR | GTGACGGGAGGAGAAACTTG | CCATCATGACAATCGGTACG | 187 |
| RK76 | SSR | TTCCATGCTTCTCCTTGTCC | CCACCATTAGGATTTTGGATG | 258 |
| RK111 | SSR | TTCCATGCTTCTCCTTGTCC | ATCGTATTGGAGGACCCCTT | 231 |
| RK69 | SSR | CTTATAGCAACCTCCGCCTG | CCAACCAACCACAAAATAAACC | 279 |
| RK34 | SSR | CTCGCCACTCTCGTTTTCTC | GCGAGTGAGATCTGGGAGAG | 190 |
| RK91 | SSR | TTGCTCGCAAGACTTCCTCT | CGTTGTTGTTGTTTTTCCAGTC | 237 |
| RK35 | SSR | GGAAACATACTTACCACTTTGACTG | GGCTTCTTGTGTGATGTTGC | 260 |
| RK38 | SSR | TCACTGTGCTTTTGAAGCTGA | GGGGTCAAAGTGAAAATATGGA | 179 |
| RK3 | SSR | GGAATTCTGGTTTGTGGTGG | AGGGTTTGCTTGGAGATCAA | 214 |
| RK82 | SSR | CAACCGCTCAGGGTATTCAT | AAGACGCAATAGGTTCATCAGA | 280 |
| RK94 | SSR | TAGCAGAGCAAACCAAGGCT | GCGTAAACGGACATCATTCA | 256 |
| RK45 | SSR | AAAATGTGTCCGGCTTTTTG | ACATTCCAACAATAGGTGGCT | 237 |
| RK47 | SSR | TTTTGGAATGGGAGAGAAGG | CAAAACCAACCTAACTCGATCA | 201 |
| RK49 | SSR | GTCGCGAGTGATAATTATAGCAAAC | AATGACGTCGTTTGTTTCCA | 249 |
| RK50 | SSR | TTTCCTGTGTCAAAGGTCCC | TGTGTCATTAATCACGAGGCA | 272 |
| RK52 | SSR | TTTCCTGTGTCAAAGGTCCC | TGTGTCATTAATCACGAGGCA | 276 |
| RK53 | SSR | AAACCTGCAGTTTCCTCTGG | TGCCTTTACGTATATCGTCTCG | 253 |
| RK58 | SSR | ATTTGCCCTAATTTCCCCAT | GAGAATGAGCGGAGACGAAC | 264 |
| RK59 | SSR | TAATTATAAGCAGCCGCCCA | CGCCTTCACCTTTCCAAGTA | 198 |
| RK64 | SSR | CATCTTCCGCCCTACTTTCA | TGGGGGTGGAGTAAGATCAG | 100 |
| RK73 | SSR | TTGTTTATTTCGACGTGCCA | TTGGAACATTTGGTCAGGAA | 150 |
| RK84 | SSR | TCCTTTCTGCTCTCTGTCCAA | CCTAGATGGCCCATTCTTCA | 153 |
| RK96 | SSR | CTCCCCAATAAAAACAACTCCA | TAGTCAACCCAACCAAAGGG | 215 |
| RK113 | SSR | CCTCAATCCCAAATAAGCCA | TTCCCATTTCCTGCTTGAAC | 271 |
| RK36 | SSR | GCGCGAGACATAACTTGTGA | GCCATGAGGGGTGAATCTAA | 198 |
| RK75 | SSR | TTAAGAGGCCGAAGCAGAAC | AGCTGCAAGTTGGCCTTATG | 176 |
| RK116 | SSR | GAAAGATCAGTTCCATAGAAAGCC | AGCAAGAGCCAATTGGAGAG | 254 |
| RK70 | SSR | TTAAACTCAGTGCGTGGTGG | CCCCAAACACCAAATCACTC | 269 |

**Table S3** SNP markers that are significantly linked with resistance to *S. endobioticum* pathotype 18. A Kruskal-Wallis test was used to calculate marker-trait associations with a p-value of 0.05 after FDR-adjustment. The physical positions of the 95 identified markers are also listed

| **SNP marker** | **Kruskal-Wallis chi-squared** | **df** | **pval** | **pvalFDR** | **chromosome** | **physical position** |
| --- | --- | --- | --- | --- | --- | --- |
| solcap_snp_c1_4319 | 115,4184021 | 1 | 6,37E-27 | 1,49E-23 | 11 | 939,591 |
| solcap_snp_c1_4322 | 115,4184021 | 1 | 6,37E-27 | 1,49E-23 | 11 | 939,924 |
| solcap_snp_c2_33740 | 107,6644968 | 1 | 3,18E-25 | 3,72E-22 | 11 | 2,029,416 |
| solcap_snp_c2_33712 | 107,6644968 | 1 | 3,18E-25 | 3,72E-22 | 11 | 2,089,302bartkS |
| solcap_snp_c2_6082 | 95,51806809 | 1 | 1,47E-22 | 1,37E-19 | 11 | 2,520,144 |
| solcap_snp_c1_2148 | 92,92310894 | 1 | 5,44E-22 | 4,24E-19 | 11 | 2,773,828 |
| solcap_snp_c2_6287 | 84,05718518 | 1 | 4,81E-20 | 2,50E-17 | 11 | 3,064,996 |
| solcap_snp_c1_2275 | 84,05718518 | 1 | 4,81E-20 | 2,50E-17 | 11 | 3,149,876 |
| solcap_snp_c2_6309 | 84,05718518 | 1 | 4,81E-20 | 2,50E-17 | 11 | 2,979,784 |
| solcap_snp_c2_37208 | 64,07456308 | 2 | 1,22E-14 | 5,71E-12 | NA | NA |
| solcap_snp_c1_4336 | 45,2480343 | 2 | 1,49E-10 | 6,36E-08 | 11 | 1,042,744 |
| solcap_snp_c2_37200 | 43,6434127 | 2 | 3,33E-10 | 1,30E-07 | 11 | 1,775,257 |
| solcap_snp_c2_6001 | 37,28567764 | 2 | 8,01E-09 | 2,88E-06 | 11 | 2,725,118 |
| solcap_snp_c2_6002 | 36,48399357 | 2 | 1,20E-08 | 4,00E-06 | 11 | 2,725,104 |
| solcap_snp_c1_2153 | 35,49002993 | 2 | 1,97E-08 | 6,13E-06 | 11 | 2,702,156 |
| solcap_snp_c1_2131 | 34,39321932 | 2 | 3,40E-08 | 9,95E-06 | 11 | 2,856,120 |
| solcap_snp_c1_4296 | 33,19098481 | 2 | 6,20E-08 | 1,61E-05 | 11 | 616,220 |
| solcap_snp_c2_13392 | 33,19098481 | 2 | 6,20E-08 | 1,61E-05 | 11 | 597,956 |
| solcap_snp_c2_6285 | 32,73823429 | 2 | 7,78E-08 | 1,92E-05 | 11 | 3,080,240 |
| solcap_snp_c2_21020 | 30,0859774 | 2 | 2,93E-07 | 6,86E-05 | 11 | 5,330,920 |
| solcap_snp_c2_21015 | 29,78375539 | 2 | 3,41E-07 | 7,59E-05 | 11 | 5,295,465 |
| solcap_snp_c2_56623 | 25,05900781 | 1 | 5,56E-07 | 7,65E-05 | 11 | 10,343,301 |
| solcap_snp_c2_56631 | 25,05900781 | 1 | 5,56E-07 | 7,65E-05 | 11 | 10,340,099 |
| solcap_snp_c2_56632 | 25,05900781 | 1 | 5,56E-07 | 7,65E-05 | 11 | 10,339,847 |
| solcap_snp_c2_32333 | 25,05900781 | 1 | 5,56E-07 | 7,65E-05 | 11 | 10,390,536 |
| solcap_snp_c2_32954 | 25,05900781 | 1 | 5,56E-07 | 7,65E-05 | 11 | 10,212,062 |
| solcap_snp_c2_33924 | 25,05900781 | 1 | 5,56E-07 | 7,65E-05 | 11 | 10,481,270 |
| solcap_snp_c2_56630 | 25,05900781 | 1 | 5,56E-07 | 7,65E-05 | 11 | 10,340,256 |
| solcap_snp_c2_56633 | 25,05900781 | 1 | 5,56E-07 | 7,65E-05 | 11 | 10,339,730 |
| solcap_snp_c2_32334 | 25,05900781 | 1 | 5,56E-07 | 7,65E-05 | 11 | 10,391,324 |
| solcap_snp_c2_44766 | 29,33165703 | 2 | 4,27E-07 | 7,65E-05 | 11 | 121,583 |
| solcap_snp_c2_44712 | 29,39869322 | 2 | 4,13E-07 | 7,65E-05 | 11 | 175,478 |
| solcap_snp_c2_20947 | 29,50338337 | 2 | 3,92E-07 | 7,65E-05 | 11 | 5,051,445 |
| solcap_snp_c2_20946 | 29,50338337 | 2 | 3,92E-07 | 7,65E-05 | 11 | 5,051,415 |
| solcap_snp_c2_13368 | 27,71829183 | 2 | 9,57E-07 | 0,00012798 | 11 | 489,973 |
| solcap_snp_c2_37580 | 22,12249213 | 1 | 2,56E-06 | 0,00030689 | 11 | 12,608,559 |
| solcap_snp_c2_33917 | 22,12249213 | 1 | 2,56E-06 | 0,00030689 | 11 | 10,852,080 |
| solcap_snp_c2_29111 | 22,12249213 | 1 | 2,56E-06 | 0,00030689 | 11 | 11,167,747 |
| solcap_snp_c2_29148 | 22,12249213 | 1 | 2,56E-06 | 0,00030689 | 11 | 11,026,810 |
| solcap_snp_c2_6000 | 25,63942859 | 2 | 2,71E-06 | 0,00030891 | 11 | 2,725,245 |
| solcap_snp_c2_21050 | 25,66831658 | 2 | 2,67E-06 | 0,00030891 | 11 | 5,464,471 |
| solcap_snp_c1_2302 | 21,53042841 | 1 | 3,48E-06 | 0,00038798 | 11 | 3,018,828 |
| solcap_snp_c2_33657 | 23,45001027 | 2 | 8,09E-06 | 0,0008802 | 11 | 2,274,063 |
| solcap_snp_c1_6148 | 19,48771445 | 1 | 1,01E-05 | 0,00107669 | 11 | 14,219,592 |
| solcap_snp_c1_6643 | 21,55293725 | 2 | 2,09E-05 | 0,0021716 | 11 | 5,515,254 |
| solcap_snp_c2_33653 | 21,14552204 | 2 | 2,56E-05 | 0,00260437 | 11 | 2,289,760 |
| solcap_snp_c2_56624 | 20,14964962 | 2 | 4,21E-05 | 0,00419386 | 11 | 10,343,292 |
| solcap_snp_c1_5936 | 16,49864526 | 1 | 4,87E-05 | 0,0044666 | 11 | 26,809,907 |
| solcap_snp_c2_18249 | 16,49864526 | 1 | 4,87E-05 | 0,0044666 | 11 | 26,809,729 |
| solcap_snp_c1_16585 | 16,49864526 | 1 | 4,87E-05 | 0,0044666 | 11 | 27,940,185 |
| solcap_snp_c1_5937 | 16,49864526 | 1 | 4,87E-05 | 0,0044666 | 11 | 26,775,893 |
| solcap_snp_c2_20989 | 15,16947605 | 1 | 9,83E-05 | 0,00884325 | 11 | 5,242,854 |
| solcap_snp_c2_4225 | 15,09514977 | 1 | 0,00010223 | 0,00890261 | 11 | 30,598,326 |
| solcap_snp_c2_5978 | 18,32983521 | 2 | 0,00010465 | 0,00890261 | 11 | 2,809,210 |
| solcap_snp_c1_2137 | 18,32983521 | 2 | 0,00010465 | 0,00890261 | 11 | 2,847,900 |
| solcap_snp_c2_6121 | 17,31949133 | 2 | 0,00017343 | 0,01409171 | 11 | 3,849,900 |
| solcap_snp_c2_47382 | 17,30512998 | 2 | 0,00017468 | 0,01409171 | 11 | 4,250,278 |
| solcap_snp_c2_6167 | 17,36951101 | 2 | 0,00016914 | 0,01409171 | 11 | 3,659,012 |
| solcap_snp_c2_4273 | 13,92651782 | 1 | 0,0001901 | 0,01482444 | 11 | 31,441,072 |
| solcap_snp_c2_20942 | 13,94394962 | 1 | 0,00018834 | 0,01482444 | 11 | 5,048,506 |
| solcap_snp_c1_6632 | 16,91869624 | 2 | 0,00021191 | 0,01625455 | 11 | 5,263,013 |
| solcap_snp_c2_4957 | 16,781542 | 2 | 0,00022695 | 0,01712757 | 11 | 20,897,824 |
| solcap_snp_c1_4005 | 13,08779422 | 1 | 0,00029723 | 0,02207494 | 11 | 31,902,799 |
| solcap_snp_c2_35868 | 12,92550275 | 1 | 0,00032414 | 0,02369735 | 11 | 7,320,658 |
| solcap_snp_c2_23958 | 12,77784048 | 1 | 0,00035075 | 0,02449489 | 11 | 6,278,396 |
| solcap_snp_c2_23945 | 12,77784048 | 1 | 0,00035075 | 0,02449489 | 11 | 6,273,163 |
| solcap_snp_c2_23947 | 12,77784048 | 1 | 0,00035075 | 0,02449489 | 11 | 6,273,331 |
| solcap_snp_c2_15642 | 12,2182714 | 1 | 0,00047324 | 0,02605034 | 10 | 55,748,590 |
| solcap_snp_c2_48146 | 12,2182714 | 1 | 0,00047324 | 0,02605034 | 10 | 55,919,092 |
| solcap_snp_c2_4245 | 15,42462942 | 2 | 0,00044728 | 0,02605034 | 11 | 6,480,977 |
| solcap_snp_c1_14231 | 12,37318232 | 1 | 0,00043554 | 0,02605034 | 10 | 55,952,532 |
| solcap_snp_c2_48128 | 12,2182714 | 1 | 0,00047324 | 0,02605034 | 10 | 55,968,047 |
| solcap_snp_c2_48092 | 12,2182714 | 1 | 0,00047324 | 0,02605034 | 10 | 56,003,386 |
| solcap_snp_c2_48130 | 12,2182714 | 1 | 0,00047324 | 0,02605034 | 10 | 55,946,835 |
| solcap_snp_c2_15654 | 12,2182714 | 1 | 0,00047324 | 0,02605034 | 10 | 55,797,725 |
| solcap_snp_c2_48127 | 12,2182714 | 1 | 0,00047324 | 0,02605034 | 10 | 55,968,104 |
| solcap_snp_c2_48145 | 12,2182714 | 1 | 0,00047324 | 0,02605034 | 10 | 55,919,521 |
| solcap_snp_c2_15641 | 12,2182714 | 1 | 0,00047324 | 0,02605034 | 10 | 55,748,465 |
| solcap_snp_c2_29151 | 12,42137079 | 1 | 0,00042445 | 0,02605034 | 11 | 11,050,480 |
| solcap_snp_c2_53688 | 12,42137079 | 1 | 0,00042445 | 0,02605034 | 11 | 9,399,582 |
| solcap_snp_c2_52749 | 12,57255416 | 1 | 0,00039145 | 0,02605034 | 11 | 7,723,697 |
| solcap_snp_c2_49296 | 12,42137079 | 1 | 0,00042445 | 0,02605034 | 11 | 9,247,353 |
| solcap_snp_c2_53272 | 12,42137079 | 1 | 0,00042445 | 0,02605034 | 11 | 9,576,187 |
| solcap_snp_c1_14553 | 12,42137079 | 1 | 0,00042445 | 0,02605034 | 11 | 9,242,943 |
| solcap_snp_c2_12276 | 12,42137079 | 1 | 0,00042445 | 0,02605034 | 11 | 8,651,904 |
| solcap_snp_c2_48087 | 11,92968934 | 1 | 0,00055246 | 0,03005789 | 10 | 55,904,334 |
| solcap_snp_c2_23988 | 11,4569584 | 1 | 0,00071227 | 0,03830691 | 11 | 6,480,977 |
| solcap_snp_c1_14475 | 11,22842972 | 1 | 0,00080554 | 0,04283081 | 10 | 58,081,119 |
| solcap_snp_c1_2212 | 14,12931081 | 2 | 0,00085479 | 0,04300602 | 11 | 3,721,765 |
| solcap_snp_c2_15616 | 11,12932996 | 1 | 0,00084973 | 0,04300602 | 10 | 55,726,339 |
| solcap_snp_c2_15622 | 11,12932996 | 1 | 0,00084973 | 0,04300602 | 10 | 55,727,260 |
| solcap_snp_c2_15637 | 11,12932996 | 1 | 0,00084973 | 0,04300602 | 10 | 55,736,946 |
| solcap_snp_c2_15632 | 11,12932996 | 1 | 0,00084973 | 0,04300602 | 10 | 55,731,584 |
| solcap_snp_c2_45475 | 10,93983571 | 1 | 0,00094119 | 0,04684899 | 11 | 10,042,367 |
| solcap_snp_c2_41086 | 10,76811407 | 1 | 0,00103264 | 0,0495379 | 11 | 33,595,761 |
| solcap_snp_c2_33926 | 10,74053365 | 1 | 0,00104814 | 0,0495379 | 11 | 10,482,154 |
| solcap_snp_c2_57107 | 10,74053365 | 1 | 0,00104814 | 0,0495379 | 11 | 10,505,722 |
| solcap_snp_c2_32338 | 10,74053365 | 1 | 0,00104814 | 0,0495379 | 11 | 10,391,657 |
| solcap_snp_c2_33906 | 10,74053365 | 1 | 0,00104814 | 0,0495379 | 11 | 10,681,117 |

**Table S4** SNP markers that are significantly linked with resistance to *S. endobioticum* pathotype 6. A Kruskal-Wallis test was used to calculate marker-trait associations with a p-value of 0.05 after FDR-adjustment. The physical positions of the 87 identified markers are also listed

| **SNP marker** | **Kruskal-Wallis chi-squared** | **df** | **pval** | **pvalFDR** | **chromosome** | **physical position** |
| --- | --- | --- | --- | --- | --- | --- |
| solcap_snp_c2_33740 | 93,0231005 | 1 | 5,17E-22 | 6,05E-19 | 11 | 2,029,416 |
| solcap_snp_c2_33712 | 93,0231005 | 1 | 5,17E-22 | 6,05E-19 | 11 | 2,089,302 |
| solcap_snp_c1_4319 | 93,7660559 | 1 | 3,55E-22 | 6,05E-19 | 11 | 939,591 |
| solcap_snp_c1_4322 | 93,7660559 | 1 | 3,55E-22 | 6,05E-19 | 11 | 939,924 |
| solcap_snp_c2_6082 | 77,2500921 | 1 | 1,51E-18 | 1,41E-15 | 11 | 2,520,144 |
| solcap_snp_c1_2148 | 73,911034 | 1 | 8,17E-18 | 6,37E-15 | 11 | 2,773,828 |
| solcap_snp_c2_6287 | 66,7382167 | 1 | 3,10E-16 | 1,61E-13 | 11 | 3,064,996 |
| solcap_snp_c1_2275 | 66,7382167 | 1 | 3,10E-16 | 1,61E-13 | 11 | 3,149,876 |
| solcap_snp_c2_6309 | 66,7382167 | 1 | 3,10E-16 | 1,61E-13 | 11 | 2,979,784 |
| solcap_snp_c2_37208 | 50,178821 | 2 | 1,27E-11 | 5,94E-09 | NA | NA |
| solcap_snp_c1_4336 | 34,9869784 | 2 | 2,53E-08 | 1,08E-05 | 11 | 1,042,744 |
| solcap_snp_c2_37200 | 34,2251667 | 2 | 3,70E-08 | 1,44E-05 | 11 | 1,775,257 |
| solcap_snp_c2_6002 | 31,4046556 | 2 | 1,52E-07 | 5,07E-05 | 11 | 2,725,104 |
| solcap_snp_c2_6001 | 31,4668099 | 2 | 1,47E-07 | 5,07E-05 | 11 | 2,725,118 |
| solcap_snp_c2_6285 | 30,0630557 | 2 | 2,96E-07 | 9,25E-05 | 11 | 3,080,240 |
| solcap_snp_c2_56623 | 24,7241589 | 1 | 6,61E-07 | 0,00012896 | 11 | 10,343,301 |
| solcap_snp_c2_56631 | 24,7241589 | 1 | 6,61E-07 | 0,00012896 | 11 | 10,340,099 |
| solcap_snp_c2_56632 | 24,7241589 | 1 | 6,61E-07 | 0,00012896 | 11 | 10,339,847 |
| solcap_snp_c2_32333 | 24,7241589 | 1 | 6,61E-07 | 0,00012896 | 11 | 10,390,536 |
| solcap_snp_c2_32954 | 24,7241589 | 1 | 6,61E-07 | 0,00012896 | 11 | 10,212,062 |
| solcap_snp_c2_33924 | 24,7241589 | 1 | 6,61E-07 | 0,00012896 | 11 | 10,481,270 |
| solcap_snp_c2_56630 | 24,7241589 | 1 | 6,61E-07 | 0,00012896 | 11 | 10,340,256 |
| solcap_snp_c2_56633 | 24,7241589 | 1 | 6,61E-07 | 0,00012896 | 11 | 10,339,730 |
| solcap_snp_c2_32334 | 24,7241589 | 1 | 6,61E-07 | 0,00012896 | 11 | 10,391,324 |
| solcap_snp_c1_4296 | 28,1986478 | 2 | 7,53E-07 | 0,00013549 | 11 | 616,220 |
| solcap_snp_c2_13392 | 28,1986478 | 2 | 7,53E-07 | 0,00013549 | 11 | 597,956 |
| solcap_snp_c1_2153 | 27,7682907 | 2 | 9,34E-07 | 0,0001618 | 11 | 2,702,156 |
| solcap_snp_c1_2131 | 27,2775857 | 2 | 1,19E-06 | 0,00019941 | 11 | 2,856,120 |
| solcap_snp_c1_6643 | 26,2641913 | 2 | 1,98E-06 | 0,00031956 | 11 | 5,515,254 |
| solcap_snp_c2_44766 | 25,9389143 | 2 | 2,33E-06 | 0,00035174 | 11 | 121,583 |
| solcap_snp_c2_44712 | 25,9600946 | 2 | 2,31E-06 | 0,00035174 | 11 | 175,478 |
| solcap_snp_c2_37580 | 21,6808514 | 1 | 3,22E-06 | 0,00043045 | 11 | 12,608,559 |
| solcap_snp_c2_33917 | 21,6808514 | 1 | 3,22E-06 | 0,00043045 | 11 | 10,852,080 |
| solcap_snp_c2_29111 | 21,6808514 | 1 | 3,22E-06 | 0,00043045 | 11 | 11,167,747 |
| solcap_snp_c2_29148 | 21,6808514 | 1 | 3,22E-06 | 0,00043045 | 11 | 11,026,810 |
| solcap_snp_c1_2302 | 21,4148542 | 1 | 3,70E-06 | 0,00048076 | 11 | 3,018,828 |
| solcap_snp_c2_21020 | 24,113734 | 2 | 5,80E-06 | 0,00073404 | 11 | 5,330,920 |
| solcap_snp_c1_6632 | 23,8383665 | 2 | 6,66E-06 | 0,00082023 | 11 | 5,263,013 |
| solcap_snp_c2_6000 | 23,5743757 | 2 | 7,60E-06 | 0,00091196 | 11 | 2,725,245 |
| solcap_snp_c1_6148 | 19,5267405 | 1 | 9,92E-06 | 0,00116041 | 11 | 14,219,592 |
| solcap_snp_c2_21015 | 22,5108348 | 2 | 1,29E-05 | 0,0014764 | 11 | 5,295,465 |
| solcap_snp_c2_20989 | 18,932297 | 1 | 1,35E-05 | 0,00150887 | 11 | 5,242,854 |
| solcap_snp_c2_20947 | 21,8297843 | 2 | 1,82E-05 | 0,00193385 | 11 | 5,051,445 |
| solcap_snp_c2_20946 | 21,8297843 | 2 | 1,82E-05 | 0,00193385 | 11 | 5,051,415 |
| solcap_snp_c1_5936 | 16,6929728 | 1 | 4,39E-05 | 0,00419615 | 11 | 26,809,907 |
| solcap_snp_c2_18249 | 16,6929728 | 1 | 4,39E-05 | 0,00419615 | 11 | 26,809,729 |
| solcap_snp_c1_16585 | 16,6929728 | 1 | 4,39E-05 | 0,00419615 | 11 | 27,940,185 |
| solcap_snp_c1_5937 | 16,6929728 | 1 | 4,39E-05 | 0,00419615 | 11 | 26,775,893 |
| solcap_snp_c2_21050 | 20,1114007 | 2 | 4,29E-05 | 0,00419615 | 11 | 5,464,471 |
| solcap_snp_c2_20942 | 16,3909228 | 1 | 5,15E-05 | 0,00482231 | 11 | 5,048,506 |
| solcap_snp_c2_13368 | 19,5511574 | 2 | 5,68E-05 | 0,00521318 | 11 | 489,973 |
| solcap_snp_c2_33657 | 19,1359803 | 2 | 6,99E-05 | 0,00629252 | 11 | 2,274,063 |
| solcap_snp_c2_4273 | 15,3101307 | 1 | 9,12E-05 | 0,0080537 | 11 | 31,441,072 |
| solcap_snp_c2_4225 | 15,2099336 | 1 | 9,62E-05 | 0,00833521 | 11 | 30,598,326 |
| solcap_snp_c2_23958 | 14,7912717 | 1 | 0,00012009 | 0,00985792 | 11 | 6,278,396 |
| solcap_snp_c2_23945 | 14,7912717 | 1 | 0,00012009 | 0,00985792 | 11 | 6,273,163 |
| solcap_snp_c2_23947 | 14,7912717 | 1 | 0,00012009 | 0,00985792 | 11 | 6,273,331 |
| solcap_snp_c2_4957 | 17,3939176 | 2 | 0,00016709 | 0,01347981 | 11 | 20,897,824 |
| solcap_snp_c2_33653 | 17,3284374 | 2 | 0,00017265 | 0,01369237 | 11 | 2,289,760 |
| solcap_snp_c2_53688 | 13,2992816 | 1 | 0,00026551 | 0,01941111 | 11 | 9,399,582 |
| solcap_snp_c2_49296 | 13,2992816 | 1 | 0,00026551 | 0,01941111 | 11 | 9,247,353 |
| solcap_snp_c2_53272 | 13,2992816 | 1 | 0,00026551 | 0,01941111 | 11 | 9,576,187 |
| solcap_snp_c1_14553 | 13,2992816 | 1 | 0,00026551 | 0,01941111 | 11 | 9,242,943 |
| solcap_snp_c2_12276 | 13,2992816 | 1 | 0,00026551 | 0,01941111 | 11 | 8,651,904 |
| solcap_snp_c1_4005 | 13,2569724 | 1 | 0,00027157 | 0,01954871 | 11 | 31,902,799 |
| solcap_snp_c2_29151 | 13,0799678 | 1 | 0,00029847 | 0,02115976 | 11 | 11,050,480 |
| solcap_snp_c2_52749 | 12,846141 | 1 | 0,00033818 | 0,02361679 | 11 | 7,723,697 |
| solcap_snp_c2_35868 | 12,6936575 | 1 | 0,0003669 | 0,02524581 | 11 | 7,320,658 |
| solcap_snp_c2_23944 | 15,4236195 | 2 | 0,00044751 | 0,03034643 | 11 | 6,263,927 |
| solcap_snp_c2_23988 | 12,0653562 | 1 | 0,00051367 | 0,0343355 | 11 | 6,480,977 |
| solcap_snp_c2_2975 | 12,0379881 | 1 | 0,00052127 | 0,03435256 | 11 | 27,834,441 |
| solcap_snp_c2_41086 | 11,7913317 | 1 | 0,00059507 | 0,03867138 | 11 | 33,595,761 |
| solcap_snp_c1_6967 | 11,7039429 | 1 | 0,00062368 | 0,0399752 | 11 | 40,777,212 |
| solcap_snp_c2_45475 | 11,6016867 | 1 | 0,00065892 | 0,04166335 | 11 | 10,042,367 |
| solcap_snp_c2_4245 | 14,5509429 | 2 | 0,00069231 | 0,04319114 | 11 | 6,480,977 |
| solcap_snp_c1_4384 | 11,4281674 | 1 | 0,00072339 | 0,04334783 | 11 | 36,265,365 |
| solcap_snp_c2_13639 | 11,4281674 | 1 | 0,00072339 | 0,04334783 | 11 | 36,658,243 |
| solcap_snp_c2_33926 | 11,3600422 | 1 | 0,00075041 | 0,04334783 | 11 | 10,482,154 |
| solcap_snp_c2_57107 | 11,3600422 | 1 | 0,00075041 | 0,04334783 | 11 | 10,505,722 |
| solcap_snp_c2_32338 | 11,3600422 | 1 | 0,00075041 | 0,04334783 | 11 | 10,391,657 |
| solcap_snp_c2_33906 | 11,3600422 | 1 | 0,00075041 | 0,04334783 | 11 | 10,681,117 |
| solcap_snp_c2_15291 | 11,2181103 | 1 | 0,00081003 | 0,04566418 | 11 | 41,722,156 |
| solcap_snp_c2_15307 | 11,2181103 | 1 | 0,00081003 | 0,04566418 | 11 | 41,686,350 |
| solcap_snp_c2_50332 | 11,1162716 | 1 | 0,00085574 | 0,04710577 | 11 | 33,927,332 |
| solcap_snp_c2_31477 | 11,1162716 | 1 | 0,00085574 | 0,04710577 | 11 | 34,059,404 |
| solcap_snp_c2_55959 | 11,0631688 | 1 | 0,0008806 | 0,04735982 | 11 | 35,093,732 |
| solcap_snp_c2_31480 | 11,0631688 | 1 | 0,0008806 | 0,04735982 | 11 | 34,568,452 |

**Table S5** Comparison of genotyping results between the 12.8 k SolCAP SNP array and KASP assay for the nine markers that were most significantly linked to resistance to *S. endobioticum* P18 and P6 in 215 dihaploid genotypes. Percentages of not available (NA) marker data for the KASP assay are also listed

| **SNP marker** | **Consistent results** | **Differing results** | **NA KASP assay** |
| --- | --- | --- | --- |
| **solcap_snp_c2_33740** | 100 % | 0 % | 0 % |
| **solcap_snp_c2_33712** | 99.08 % | 0 % | 0.91 % |
| **solcap_snp_c1_4319** | 98.63 % | 0 % | 1.37 % |
| **solcap_snp_c1_4322** | 99.08 % | 0 % | 0.91 % |
| **solcap_snp_c2_6082** | 96.35 % | 0.91 % | 2.74 % |
| **solcap_snp_c2_6287** | 97.72 % | 0 % | 2.28 % |
| **solcap_snp_c1_2275** | 99.08 % | 0 % | 0.91 % |
| **solcap_snp_c2_6309** | 96.35 % | 0 % | 3.65 % |
| **solcap_snp_c2_6285** | 99.08 % | 0 % | 0.91 % |

**Table S6** Marker-trait association of the most significant markers linked to resistance to *S. endobioticum* P18. For each marker, the number of recombinant genotypes, the p-value of the Kruskal-Wallis test with and without FDR adjustment, and the mean resistance scores of the groups with and without markers are listed

| **Marker** | **Number of recombinant genotypes** | **p-value Kruskal-Wallis** | **p-value after FDR adjustment** | **Mean marker present** | **Mean marker absent** |
| --- | --- | --- | --- | --- | --- |
| solcap_snp_c1_4322  solcap_snp_c1_4319 | 1 | 6,37E-27 | 1,49E-23 | 2.063676 | 3.799902 |
| SSCP4348  Kc8103  RK7  RK75  RK76  RK70  RK69  RK91 | 0 | 8,44E-28 | 3,96E-24 | 2.014091 | 3.797981 |
| RK36  SSCP13  SSCP14  SSCP15 | 1 | 5,16E-27 | 1,21E-23 | 2.015846 | 3.779905 |
| Y1delATT | 2 | 4,60E-26 | 4,31E-23 | 2.045909 | 3.777788 |
| solcap_snp_c2_33740  solcap_snp_c3_33712 | 3 | 3,18E-25 | 3,72E-22 | 2.076567 | 3.77466 |

**Fig. S1** Scheme of the experimental approach conducted in this study
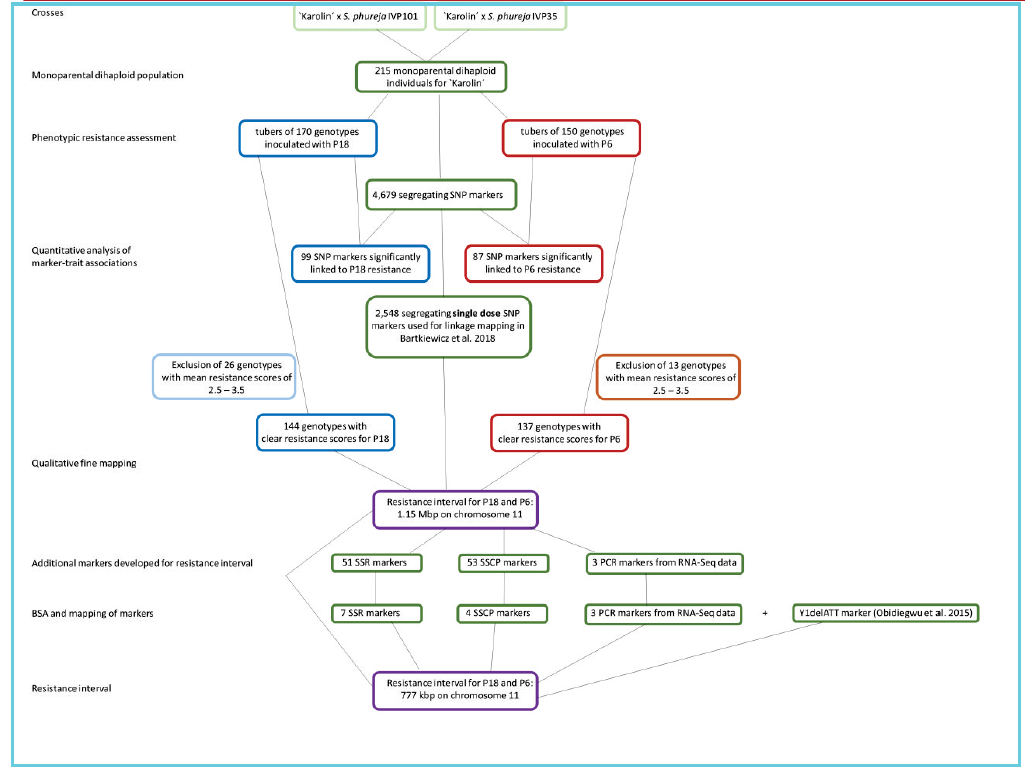


**Fig. S2** Agarose gel electrophoresis of PCR marker Kc8103 in 16 tetraploid potato varieties with known resistance to *S. endobioticum* P18. Resistant varieties show a marker band at 2197 bp, which is not present in the susceptible varieties. Varieties `Jutrzenka´ and `Saphir´ as well as `Merano´ and `Milek´ show non-matching genotypes for this marker


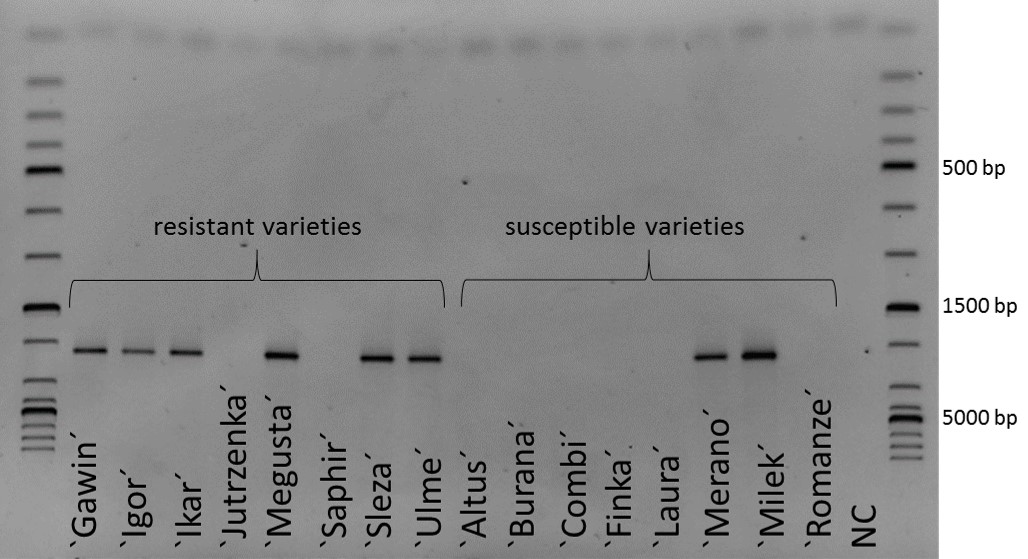

Supplement: Supplementary file 1 — Supplementary material 1 (DOCX 382 kb) [file 122_2018_3172_MOESM1_ESM.docx]
